# Identification of Novel Aldose Reductase Inhibitors from Spices: A Molecular Docking and Simulation Study

**DOI:** 10.1371/journal.pone.0138186

**Published:** 2015-09-18

**Authors:** Priya Antony, Ranjit Vijayan

**Affiliations:** Department of Biology, College of Science, United Arab Emirates University, PO Box 15551, Al Ain, Abu Dhabi, United Arab Emirates; University of Akron, UNITED STATES

## Abstract

Hyperglycemia in diabetic patients results in a diverse range of complications such as diabetic retinopathy, neuropathy, nephropathy and cardiovascular diseases. The role of aldose reductase (AR), the key enzyme in the polyol pathway, in these complications is well established. Due to notable side-effects of several drugs, phytochemicals as an alternative has gained considerable importance for the treatment of several ailments. In order to evaluate the inhibitory effects of dietary spices on AR, a collection of phytochemicals were identified from *Zingiber officinale* (ginger), *Curcuma longa* (turmeric) *Allium sativum* (garlic) and *Trigonella foenum graecum* (fenugreek). Molecular docking was performed for lead identification and molecular dynamics simulations were performed to study the dynamic behaviour of these protein-ligand interactions. Gingerenones A, B and C, lariciresinol, quercetin and calebin A from these spices exhibited high docking score, binding affinity and sustained protein-ligand interactions. Rescoring of protein ligand interactions at the end of MD simulations produced binding scores that were better than the initially docked conformations. Docking results, ligand interactions and ADMET properties of these molecules were significantly better than commercially available AR inhibitors like epalrestat, sorbinil and ranirestat. Thus, these natural molecules could be potent AR inhibitors.

## Introduction

**Fig 1:**
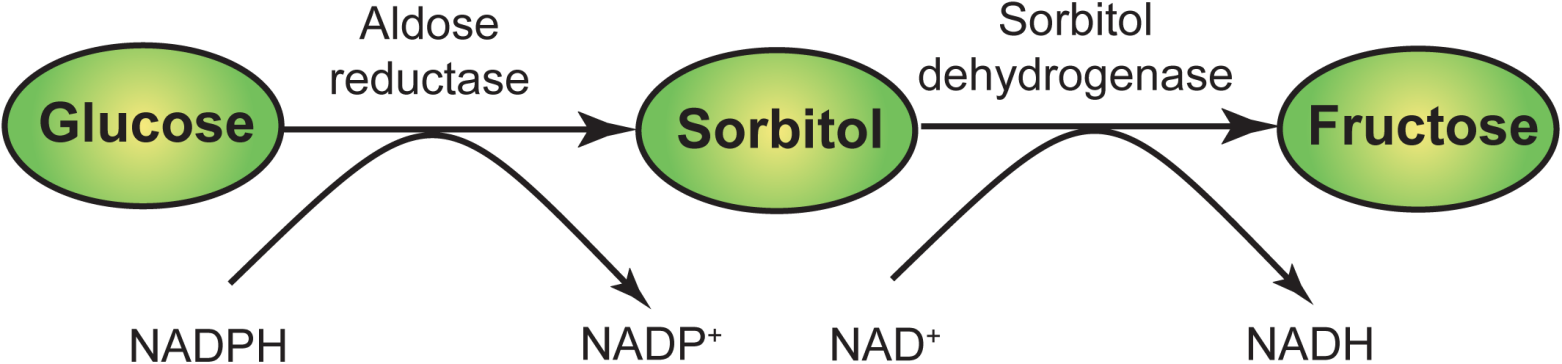
Polyol pathway.

Diabetes mellitus is a complex metabolic illness characterized by elevated levels of blood glucose. It is a major health threat that is rapidly growing globally. An International Diabetes Federation (IDF) estimate indicated that over 387 million people are living with diabetes and this is expected to reach 592 million or more by 2035 [1]. A serious issue in diabetes is the gradual development of complications in insulin independent tissues such as nerves, retina, lens glomerulus and vascular cells [2]. Increased oxidative stress and aldose reductase (AR) activity is thought to play a pivotal role in complications such as diabetic neuropathy, retinopathy, cardiomyopathy, nephropathy, cataracts, myocardial infarctions and even stroke [3–4]. One of the most studied biochemical pathway associated with hyperglycemia is the polyol pathway (Fig 1). This is a two-step pathway in which AR is the major rate limiting enzyme [5]. It reduces glucose to the alcohol sorbitol using NADPH as a cofactor. Sorbitol is subsequently metabolized to fructose by the enzyme sorbitol dehydrogenase using NAD^+^ as a cofactor [6] (Fig 1).

In normal glycemic conditions, most of the cellular glucose is channelled through the glycolytic pathway; only minor amount of non-phosphorylated glucose enters the polyol pathway. However, under hyperglycemic conditions, more than 30% of glucose is metabolized through the polyol pathway, which in turn generates oxidative stress in cells [7]. Multiple mechanisms have been proposed to explain how the polyol pathway induces oxidative stress and tissue damage. Firstly, an increased consumption of NADPH could reduce the amount of NADPH available to the enzyme glutathione reductase (GR). GR uses NADPH as a cofactor for the generation of glutathione (GSH). GSH is an important scavenger of reactive oxygen species (ROS). Thus, NADPH depletion reduces the cellular capability to withstand oxidative stress. Next, NAD^+^ is converted to NADH by sorbitol dehydrogenase (SDH). This increases NADH ratio which is utilized by NADH oxidase leading to the production of reactive oxygen species (ROS) that could attack mitochondrial membranes. Lastly, the fructose produced in this pathway is metabolized to fructose-3-phosphate and 3-deoxyglucosone. Both compounds are potent glycosylating agents which results in the formation of advanced glycation end products (AGE) [8–9]. Moreover, intracellular sorbitol accumulation also promotes osmotic and oxidative stress [10]. Thus, increased glucose flux through the polyol pathway increases cellular susceptibility to oxidative stress in a number of different ways. The key regulator of this pathway is AR, a small monomeric protein belonging to the aldo-keto reductase superfamily. It consists of a β/α-barrel structural motif with a large hydrophobic active site [11]. The significant role of AR in hyperglycemic conditions has been ascertained in several biochemical and cellular studies. Highly overexpressed AR and increased level of sorbitol was observed in mouse Schwann cells during hyperglycemic condition [12]. Increased sorbitol accumulation and AR activity was also reported in diabetic patients [13]. Moreover, increased AR activity contributes to oxidative stress and cataract formation in retina [14, 15] and inhibition of AR improves the glucose metabolism in the heart of diabetic rats[16]. These observations suggest that AR could play a significant role in long term diabetic complications. Thus, inhibition of AR is a potential treatment for diabetic complications. Currenlty, the main types of AR inhibitors are carboxylic acid inhibitors (e.g. epalrestat), spirohydantoin derivatives (e.g. sorbinil) and succinimide compounds (e.g. ranirestat) [17–18]. A large number of molecules have been designed and synthesized to inhibit AR. However, only a limited number of drugs have reached the market [19]. At the moment, epalrestat is the only AR inhibitor which in available in markets like India and Japan. Some drugs were withdrawn due to safety concerns and others are still in clinical trials [20]. Thus, it is very crucial to develop new AR inhibitors with improved efficacy and safety profile.

History of using plants, herbs and spices as medicines dates back to ancient times. Plants are rich sources of active principles and a vast majority of currently available therapeutic drugs were derived directly or indirectly from plants [21]. Spices are dried seeds, fruits, barks, leaves or even roots of plants that are used as flavouring, colouring and preservative agents. The medicinal properties of spices have been well appreciated in preventing various ailments such as diabetes, cancer, inflammations, cardiovascular diseases, etc. [22]. The aim of the current study was to identify any potential AR inhibitors that may be present in spices such as ginger, turmeric, garlic and fenugreek. Ginger is the rhizome part of plant *Zingiber officinale* which contains many bioactive principles such as gingerols, gingerenones and shogaols. A number of *in vitro* and *in vivo* studies have examined the efficacy of ginger in controlling diabetes [23–27]. Methanolic extract of garlic (*Allium sativum*) possess hypoglycemic properties and delays the progression of diabetic complications [28–29]. Administration of turmeric (*Curcuma longa*) in diabetic rats significantly lowered blood glucose level and reduces hyperglycemia induced oxidative stress [30–31]. *Trigonella foenum-gracecum*, commonly known as fenugreek, was also extensively studied for its hypoglycemic effects. Antioxidant potential of fenugreek seed extract was shown to reduce the oxidative stress developed during diabetic conditions [32–33]. The exact mechanism of action of these spices on the polyol pathway and various diabetic complications remain elusive. Therefore, the present study was designed to investigate the effect of these spices, or more specifically the phytochemicals present in these spices, on AR, the key regulator of the polyol pathway. Computational methods including molecular docking and molecular dynamics (MD) simulations were employed to study the binding modes and interactions of a collection of phytochemicals to AR. This provided detailed insights into how some of the selected phytochemicals interact with AR possibly assisting in lead identification and further drug development.

## Materials and Methods

### Pre-processing of targets and ligands

Three high-resolution X-ray crystal structures of human AR (PDB IDs 4GCA [34], 4LAU [35], 1US0 [36]) with resolutions 0.90 Å, 0.84 Å, 0.66 Å were retrieved from the Protein Data Bank [37]. Three AR structures were used so that the results were not biased by a single structure. This ensures the validity of the results in the instance where the structure and size of the active site are different. The active site of AR has a volume of 312.473 Å^3^ and is complexed with NADP^+^ and IDD1219 in the structure with PDB ID 4GCA. The active site volume is 299.096 Å^3^ and complexed with NADP^+^ and {2-[(4-bromobenzyl)carbamoyl]-5-chlorophenoxy}acetic acid in the structure with PDB ID 4LAU, while the active site volume is 298.753 Å^3^ and complexed with the molecules NADP^+^ and IDD 594 in the structure with PDB ID 1US0.

The retrieved structures were pre-processed in Schrödinger Maestro [38]. This step included simplification of multimeric structures, proper assignment of bond orders and ionization states, addition and optimization of hydrogen bonds, location and deletion of unnecessary water molecules, creation of disulphide bonds, conversion of selenomethionines to methionine, aligning and capping of terminal amides, addition of missing atoms and side chain residues, and assignment of partial charges. Finally, restrained minimization was performed to obtain a geometrically stable structure [39]. A ligand library, consisting of 212 phytochemicals from the 4 spices mentioned above (S1 Table), was prepared using 2D structures obtained from PubChem and ChemSpider databases [40] as the starting point. For a comparative analysis, 2D structures of the drug molecules epalrestat, sorbinil and ranirestat were also obtained from PubChem. Drug molecules and phytochemicals were pre-processed and conformers were generated using Schrödinger Ligprep [41]. Pre-processing of ligands included conversion of 2D structures to 3D format, addition of hydrogen atoms, generation of tautomer and ionization states, neutralization of charged groups, structure filtration and finally geometry optimization of the molecule.

### Active site identification and grid generation

Based on the NADPH cofactor binding location, the active site of the AR is located at carboxyl terminal end of the beta barrel [42]. Nonetheless, binding site detection was performed which showed that AR consists of only one druggable binding pocket as identified above. The amino acid residues that are directly involved in the ligand binding were identified from the literature [43]. The active site consists of anion binding region and specificity region. The anion binding region is rigid and occupied by amino acids such as Trp 20, Val47, Tyr 48, Trp79, His 110, and Trp111 [44]. The specificity pocket, which shows high range of selectivity and flexibility, is lined by Trp111, Thr113, Phe122, Gln183, Trp 209, Cys 298, Leu 300, and Cys 303 [17, 45]. Therefore a receptor grid was generated incorporating all these functional residues.

### Standard precision (SP) and extra precision (XP) docking

For predicting the binding orientation, affinity and activity of ligand molecules with the targets, molecular docking was employed [46]. Grid based docking was carried out using Schrödinger Glide [47]. Standard precision (SP) docking was performed followed by extra precision (XP) docking [48]. To soften the potential of nonpolar parts of ligands, the scaling factor for the ligand van der Waals radii was set in 0.80 with a partial atomic charge of 0.15. In both SP and XP docking procedures, flexible ligand sampling was used to generate various ligand conformations. No constraints were used in the entire docking studies. OPLS 2005 force field was used and post docking minimization was also performed. All phytochemicals and drug molecules were docked flexibly to the three structures of the enzyme AR.

### Analysis and visualization of docking results

After docking, top ranked compounds were arranged based on the GlideScore [49]. Lower GlideScore represents more favourable binding. Hydrogen bond interactions, π interactions and hydrophobic interactions of the best poses were visualized and interpreted using XP visualizer and PyMol [50]. Binding energy based on molecular mechanics generalized Born surface area (MM-GBSA) was calculated using Schrödinger Prime [51].

### Molecular dynamics (MD) simulations

In order to explore the stability and variability of top ranking docked complexes, MD simulations were performed [52]. Two top scoring protein-ligand complexes were simulated using Desmond. Each protein-ligand complex, represented using the OPLS 2005 forcefield [53], was solvated in an orthorhombic box of single point charge (SPC) water molecules [54] with a box wall distance of 10 Å. The system was neutralized by adding the required number of counter ions and a salt concentration of 0.15M was used.

The default six-stage system relaxation protocol was employed before starting production runs. This consisted of a series of minimization and equilibration simulations to slowly relax the system while not significantly deviating from the initial conformation. The first two stages consisted of 2000 steps of steepest descent minimization with and without a restraint of 50 kcal/mol/Å^2^ on the solute atoms. Four short MD simulations were then performed: (i) 12 ps MD simulation in NVT (constant number of particles, volume and temperature) ensemble at 10 K with solute heavy atoms restrained with a force constant of 50 kcal/mol/Å^2^; (ii) 12 ps MD simulation in NPT (constant number of particles, pressure and temperature) ensemble at 10 K with the same restraint; (iii) 12 ps MD simulation in NPT ensemble at 300 K with the same restraint; (iv) 24 ps MD simulation in NPT ensemble at 300 K without any restaints. Unrestrained production simulations were then performed in the NPT ensemble for 100 ns at 300 K temperature and 1.01325 bar pressure. For this, the Nosé-Hoover chain thermostat [55] was used with a relaxation time of 1 ps and the isotropic Martyna-Tobias-Klein barostat [56] was used with a relaxation time of 2 ps. A cutoff of 9 Å was used for evaluating short range interactions, while long range coulombic interactions were evaluated using the smooth particle mesh Ewald method (PME) [57]. The RESPA integrator [58] was used with an inner time step of 2 fs and an outer time step of 6 fs. Simulation trajectories were saved by capturing frames every 4.8 ps. After simulations, root mean square deviations (RMSD), root mean square fluctuations (RMSF), and protein-ligand contacts of the complexes were calculated. Rescoring of the protein-ligand interactions was perfomed using Glide with frames extracted every 5 ns from the 100 ns MD simulations.

### Pharmacokinetic parameters

Drug likeliness and ADME studies of ligands was performed to eliminate unfavourable compounds in the early stage of drug development process. Schrodinger Qikprop [59] was used for predicting the pharmacokinetic properties of the selected ligands. Using Qikprop, the Lipinski’s rule of five and various principal parameters such as absorption, metabolism, aqueous solubility, blood-brain barrier penetration, and central nervous system (CNS) activity were calculated [60].

## Results and Discussions

### Validation of molecular docking protocol

One of the most widely used methods for validating a docking protocol is the re-docking of co-crystalized ligand to the target protein [61]. In this study, the co-crystallized ligands were extracted from the three receptor proteins and re docked into the active site of the respective receptor. After docking, the best pose of the ligand was aligned with the co-crystalized ligand and RMSD of the ligand was calculated. This helps to determine the reliability and reproducibility of the docking protocol [62]. All docked conformations of ligands when superimposed with respective co-crystallized ligands were within an RMSD of 2.0 Å indicating a valid docking protocol.

### Standard precision and extra precision docking

**Fig 2:**
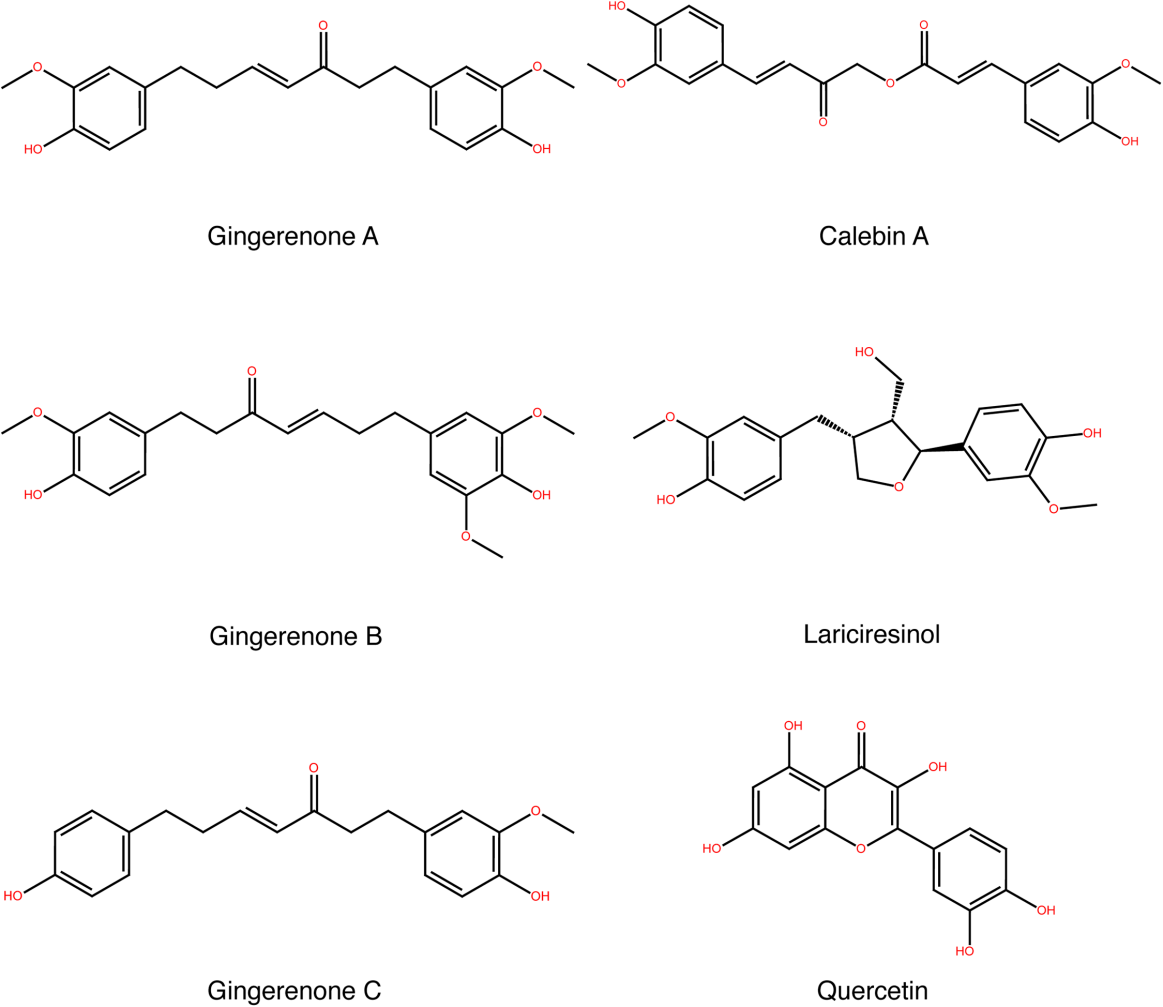
Chemical structure of lead molecules.

To confirm the validity of the results, docking was performed with three structures of AR (PDB IDs: 4GCA, 4LAU, 1US0). All ligand conformers were flexibly docked into the selected AR X-ray crystal structures. Initially, SP docking method was employed and the highest scoring compounds were subjected to XP docking. After XP analysis, the best interacting compounds were ranked based on GlideScore and the best pose of the ligand was chosen. The top ranked compounds that docked to AR are shown in Table 1 along with the drug molecules epalrestat, sorbinil and ranirestat. For comparison, SP GlideScore for these compounds are provided in S2 Table. Out of the 212 natural compounds selected initially, gingerenone A, gingerenone B, quercetin, lariciresinol, gingerenone C and calebin A showed high GlideScores (Table 1) and binding energies (Table 2). The chemical structures of these lead compounds are shown in Fig 2. These compounds interacted with the target proteins by forming hydrogen bonds, hydrophobic interactions and π–π interactions with the active site residues including Trp20, Val47, Tyr48, Trp79, His110, Trp111, Thr113, Phe122, Gln183, Tyr209, Ala299, and Leu300. These are crucial amino acid residues that are directly involved in the binding of an inhibitor.

**Table 1:** GlideScore and interacting residues from XP docking of various phytocompounds and drugs on AR targets.

Target	Ligand	XP GlideScore (kcal/mol)	Hydrogen bonding residues	Residues forming π–π interactions
4GCA	Gingerenone A	-13.02	Thr113, Gln183	Trp20, His110, Trp111, Tyr209
4GCA	Gingerenone B	-11.87	Thr113	Trp20, His110, Trp111, Tyr209
4GCA	Quercetin	-11.87	Leu300, His110	Trp20, His110, Trp111
4GCA	Lariciresinol	-11.77	Gln183	Trp20, His110, Trp111, Tyr209
4GCA	Calebin A	-11.76	Thr113, Gln183	Trp20, His110, Trp111, Tyr209
4GCA	Gingerenone C	-11.41	Thr113, Gln183	Trp20, His110, Trp111, Tyr209
4GCA	Ranirestat	-9.7	Trp111, Tyr48	Nil
4GCA	Epalrestat	-9.5	Trp20	Trp111
4GCA	Sorbinil	-7.4	Tyr48, His110	Nil
4LAU	Gingerenone B	-11.92	Trp111, Gln183	Trp20, His110, Trp111, Tyr209
4LAU	Gingerenone A	-11.66	Thr113, Gln183	His110, Trp111, Tyr209
4LAU	Quercetin	-11.55	Thr113	Trp79,Trp111
4LAU	Calebin A	-11.13	Thr113, Asn160, Ala299	Trp20, His110, Trp111
4LAU	Lariciresinol	-11.05	Gln183	Trp20, His110, Trp111, Tyr209
4LAU	Gingerenone C	-10.68	Tyr48, His110, Thr113	Trp111
4LAU	Epalrestat	-9.7	Trp20	Trp111
4LAU	Ranirestat	-9.2	Tyr48, His110	Trp111
4LAU	Sorbinil	-7.7	Trp20, Trp111	Phe122
1US0	Gingerenone B	-11.63	Tyr48, His110	Trp111
1US0	Gingerenone A	-11.77	Thr113, Gln183	His110, Trp111, Tyr209
1US0	Quercetin	-11.53	Thr113	Trp79, Trp111, Phe122
1US0	Gingerenone C	-11.50	Thr113, Gln183	His110, Trp111, Tyr209
1US0	Lariciresinol	-11.48	No H bonds	His110, Trp111, Tyr209
1US0	Calebin A	-11.48	Thr113, Gln183	Trp20, His110, Trp111, Tyr209
1US0	Ranirestat	-9.7	Trp111	Trp111
1US0	Epalrestat	-9.4	Trp20	Trp111
1US0	Sorbinil	-8.7	Trp111	Trp111

**Table 2:** Binding energy calculated using MM-GBSA approach. ΔG_Binding energy_ is the sum of the other columns.

Target	Ligand	ΔG_Binding energy_ (kcal/mol)	ΔG_Coulomb energy_ (kcal/mol)	ΔG_Covalent binding energy_ (kcal/mol)	ΔG_Hydrogen-bonding energy_ (kcal/mol)	ΔG_Lipophilic energy_ (kcal/mol)	ΔG _Van der Waals energy_ (kcal/mol)	ΔG_Pi-pi stacking energy_ (kcal/mol)	ΔG _Generalized Born electrostatic solvation energy_ (kcal/mol)
4GCA	Gingerenone A	-95.01	-13.90	4.49	-0.70	-49.57	-49.91	-5.92	20.50
4GCA	Gingerenone B	-73.16	-26.27	18.68	0.68	-47.80	-45.86	-3.63	32.42
4GCA	Quercetin	-59.00	-19.89	14.32	-0.79	-28.79	-39.45	-6.57	22.19
4GCA	Lariciresinol	-79.12	-12.00	7.78	-0.31	-50.44	-37.29	-6.02	19.16
4GCA	Calebin A	-91.61	-10.12	8.41	-0.55	-45.03	-53.45	-6.97	16.11
4GCA	Gingerenone C	-100.93	-20.06	7.35	-0.64	-51.30	-50.66	-5.88	20.28
4GCA	Ranirestat	-70.06	-3.65	6.02	-0.62	-37.26	-36.96	-1.81	4.25
4GCA	Epalrestat	-62.57	-2.51	13.01	-1.09	-42.04	-49.66	-2.77	22.50
4GCA	Sorbinil	-48.33	-8.63	1.01	-0.63	-17.72	-25.09	-0.84	3.57
4LAU	Gingerenone B	-76.74	-11.36	14.15	-0.72	-56.91	-32.50	-4.26	14.86
4LAU	Gingerenone A	-88.19	-13.59	10.46	-0.67	-55.89	-41.99	-7.61	21.10
4LAU	Quercetin	-60.52	-12.89	3.06	-0.48	-29.11	-35.62	-11.67	26.20
4LAU	Calebin A	-85.78	-9.36	9.19	-0.86	-53.43	-41.0	-4.17	13.88
4LAU	Lariciresinol	-85.51	-4.83	6.88	-0.27	-48.82	-49.45	-7.05	18.03
4LAU	Gingerenone C	-76.61	-10.35	0.26	-0.54	-39.32	-41.87	-4.51	19.73
4LAU	Epalrestat	-68.57	5.29	5.77	-1.00	-41.68	-46.05	-3.39	12.51
4LAU	Ranirestat	-88.03	-9.22	5.10	-0.60	-37.80	-42.86	-5.56	2.91
4LAU	Sorbinil	-37.67	-1.63	0.72	-0.41	-20.04	-23.50	-0.06	7.25
1US0	Gingerenone B	-88.78	-34.85	21.04	-0.93	-59.319	-38.72	-5.23	29.22
1US0	Gingerenone A	-89.44	-13.22	5.99	-0.55	-53.47	-46.55	-6.03	24.40
1US0	Quercetin	-65.79	-15.94	3.650	-0.506	-28.84	-39.55	-11.81	27.22
1US0	Gingerenone C	-85.12	-15.03	2.246	-0.735	-45.05	-42.87	-4.86	21.18
1US0	Lariciresinol	-91.00	-11.55	11.51	-0.54	-51.45	-49.87	-3.36	14.27
1US0	Calebin A	-86.12	-20.44	4.6	-0.78	-38.01	-47.97	-4.75	21.01
1US0	Ranirestat	-74.73	4.32	5.03	-0.33	-39.42	-46.57	-4.85	7.09
1US0	Epalrestat	-68.38	-7.14	4.17	-1.02	-41.00	-45.48	-3.12	25.23
1US0	Sorbinil	-47.16	-2.18	3.09	-0.18	-25.14	-28.93	-5.39	11.58

### Interaction analysis of phytocompounds with AR

From Tables 1 and 2, out of the six compounds, gingerenones A and B exhibited high binding score and binding energy towards AR. Gingerenones are diarylheptanoids seen in the rhizome of plant Z*ingiber officinalae* and the basic structure consist of two aromatic rings connected by a heptane chain [63]. Only the antifungal property of gingerenones has been reported so far [64]. Due to the hydrophobic nature of active site, aromatic compounds serve as the best substrate for this enzyme [65]. In agreement with this, π–π stacking also contributes to the binding energy in these compounds apart from the high lipophilic and van der Waals’ contributions (Table 2). Quercetin also showed a high docking score. In fact quercetin has already been reported as a strong AR inhibitor [66]. In the present study, gingerenones A and B showed better GlideScore and binding energy than quercetin. However, calebin A, lariciresinol and gingerenone C exhibited lower GlideScore compared to natural AR inhibitor quercetin.

**Fig 3:**
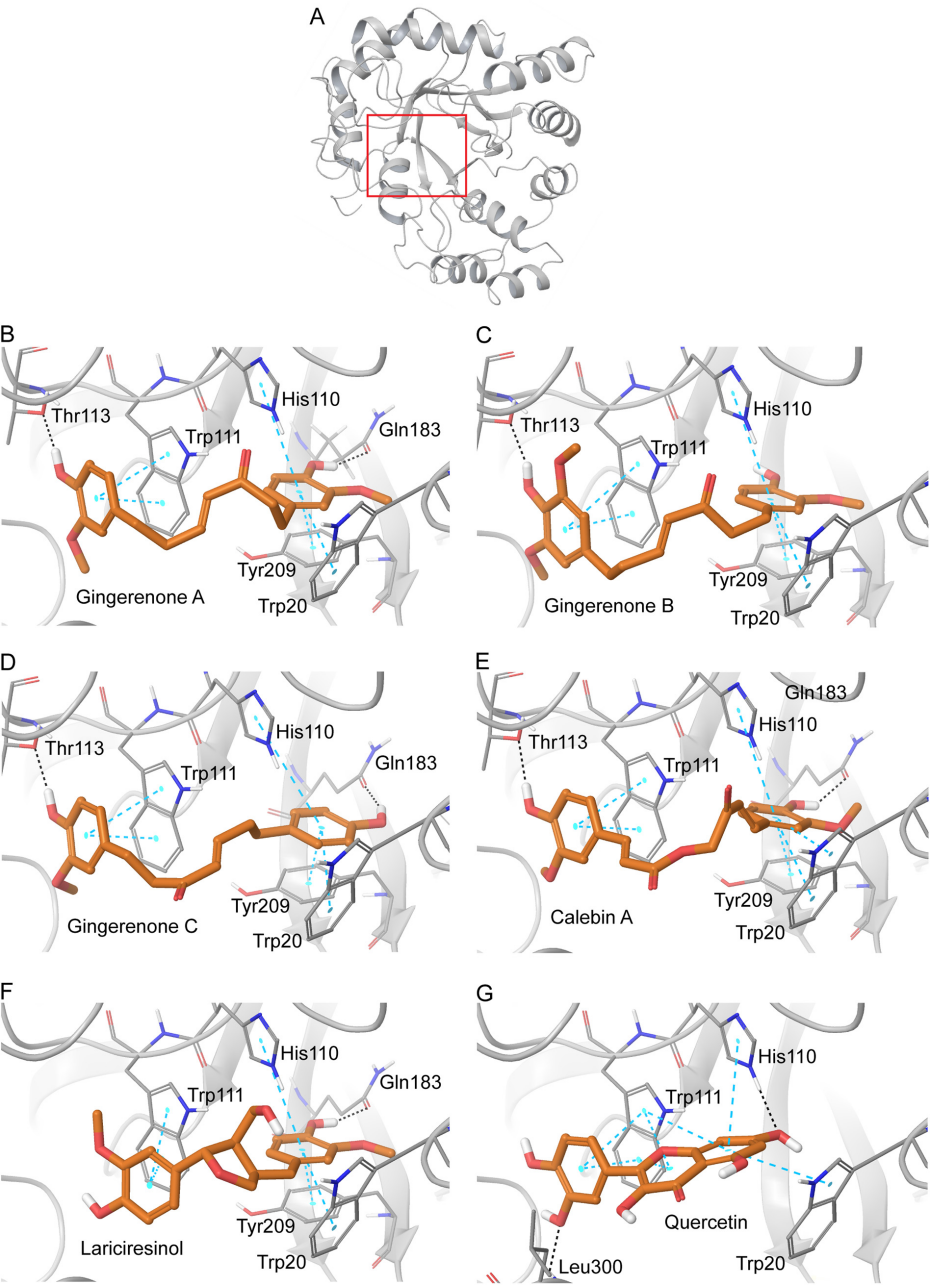
Molecular interactions of lead compounds with AR (PDB ID: 4GCA). (A) Structure of AR with the binding site region enclosed in a red box. (B) AR-gingerenone A complex (C) AR-gingerenone B complex (D) AR-gingerenone C complex (E) AR-calebin A complex (F) AR-lariciresinol complex (G) AR-quercetin complex. Protein is shown in grey cartoon representation, amino acid side chains are shown in stick representation and the docked ligand is in orange. Hydrogen bonds are shown as black dotted lines and π–π interactions are shown as blue lines.

**Fig 4:**
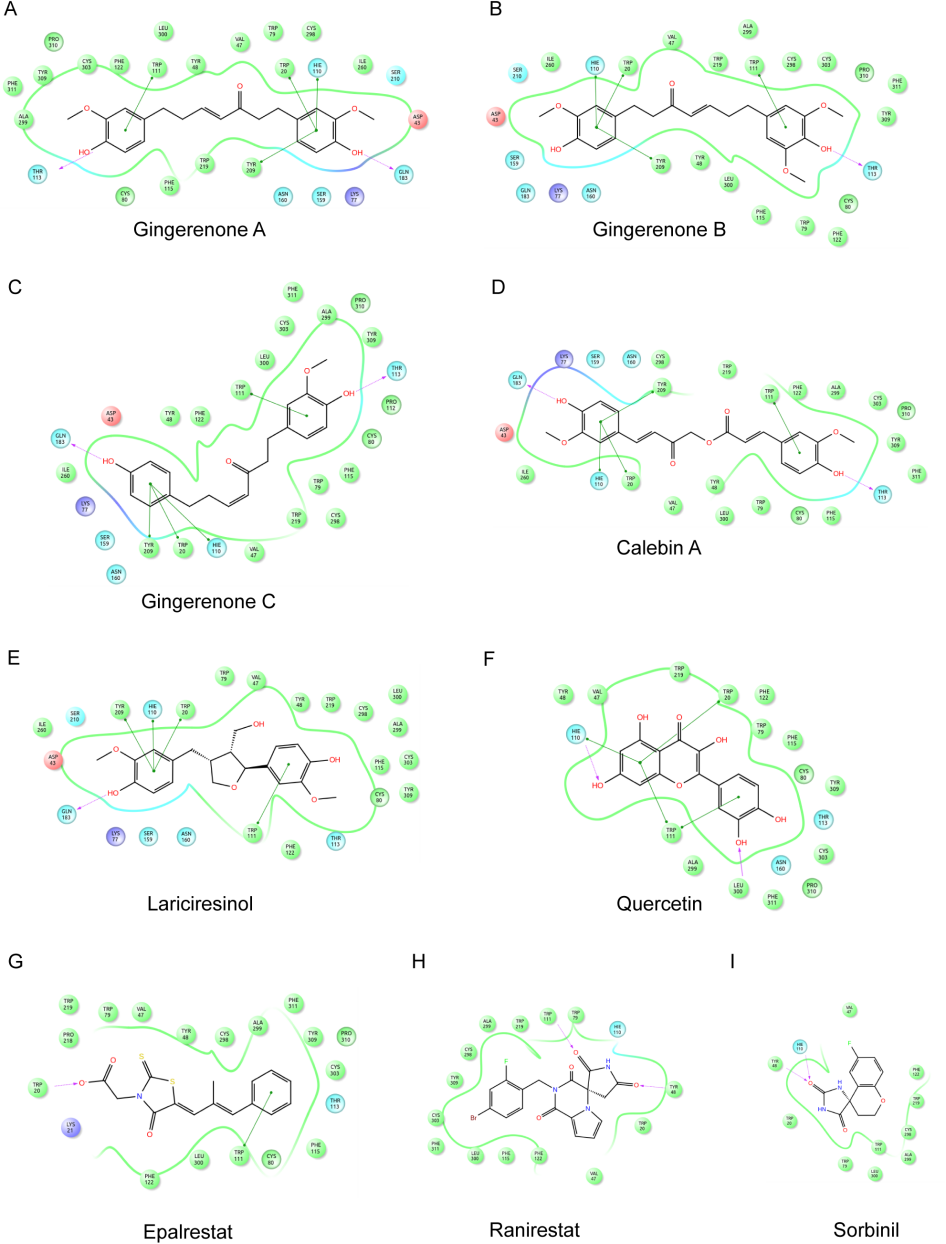
Ligand interaction diagram of lead compounds with AR (PDB ID: 4GCA). (A) AR-gingerenone A complex (B) AR-gingerenone B complex (C) AR-gingerenone C complex (D) AR-calebin A complex (E) AR-lariciresinol complex (F) AR-quercetin complex. Colored circles indicate amino acids that interact with the bound ligand. Negatively charged amino acids are represented with red circles, positively charged amino acids are represented with dark blue circles, polar amino acids are represented with light blue circles and hydrophobic amino acids are represented with green circles. Hydrogen bonds are represented with purple arrows–dashed arrows for hydrogen bonds involving amino acid side chain and regular arrows for hydrogen bonds involving amino acid backbone. π–π interactions are shown with green lines.

In the case of 4GCA-gingerenone A complex (Figs 3B and 4A), the ligand interacted with the protein by forming two hydrogen bonds, four π–π interactions and several hydrophobic contacts. The two aromatic rings of gingerenone A are firmly anchored in the anionic binding site by the formation of π–π interactions with Trp20, His110, Trp111, and Tyr209. Furthermore, it forms hydrogen bonds with Thr113 (1.73 Å) and Gln183 (1.85 Å). The residues Trp111 and Gln183 are part of the specificity pocket [67]. By interacting with these residues gingerenone A show higher selectivity for AR [68]. Moreover it possesses a high GlideScore of -13.02 kcal/mol and binding energy of -95.01 kcal/mol. Likewise, in 4LAU-gingerenone A complex (S1 and S4 Figs), the ligand is bound to the active site by forming hydrogen bonds, hydrophobic interactions and π–π interactions. In 1US0-gingerenone A complex (S2 and S5 Figs), two hydrogen bonds three π–π interactions and numerous hydrophobic interactions were observed. The mode of binding and interaction of residues were also similar to 4GCA and 4LAU.

In 4GCA-gingerenone B complex (Figs 3C and 4B), one hydrogen bond, four π–π interactions and numerous hydrophobic interactions were observed. Thr113 formed a hydrogen bond (1.96 Å) and Trp20, His110, Trp111, and Tyr209 were involved in π–π interactions. These residues were seen in the anion binding site and specificity region of the active site [64]. Gingerenone B exhibited a similar binding mode as that of gingerenone A as both the aromatic rings were bound to the active site by the formation π–π stacking interactions. In 4LAU (S1 and S4 Figs), the ligand gingerenone B exhibited the same interactions by forming hydrogen bonds and π–π stacking with Trp20, His110, Trp111, Gln183, and Tyr209. In 1US0 (S2 and S5 Figs), the ligand forms hydrogen bonds with hydrophilic residues such as Tyr48 (2.77Å) and His110 (1.71Å). The aromatic ring forms a π–π stacking interaction with Trp111. In the three protein structures, gingerenone B exhibited a GlideScore ranging from -11.63 to -11.92 kcal/mol. Thus, in the three protein structures used in this study, gingerenones A and B showed a high affinity towards AR.

Lariciresinol docked into 4GCA and 4LAU (Figs 3F and 4E, S1 and S4 Figs) by the formation of one hydrogen bond and four π–π interactions. The residues involved in these interactions are Trp20 and His110 Trp111, Tyr209, and Gln183. In 1US0 (S2 and S5 Figs), lariciresinol forms π–π interaction with Trp20, His110, and Trp111. In the three AR target structures, lariciresinol exhibited a GlideScore of -11.77, -11.05, and -11.48 kcal/mol respectively. Calebin A, the phytochemical present in *Curcuma longa* produced interactions with structures 4GCA (Figs 3E and 4D) and 1US0 (S2 and S5 Figs) by forming hydrogen bonds and π–π stacking with Trp20, His110, Trp111, Thr113, Gln183, and Tyr 209. In 4LAU (S1 and S4 Figs), calebin A formed hydrogen bonds with Thr113, Asn160, and Ala 299 which are seen in the specificity pocket of the active site. Trp20, His110, and Trp111 were involved in the π–π interactions with target protein. Gingerenone C is another diarylheptanoid present in *Zingiber officinalae*. It interacted with AR structures by forming hydrogen bonds, hydrophobic interactions and π–π interactions (Figs 3D and 4C, S1, S2, S4 and S5 Figs). The residues involved in forming hydrogen bonds are Tyr48, His110, Thr113, and Gln183. These are residues seen in both anion binding pocket and specificity pocket. Trp20, His110, Trp111, and Tyr209 were involved in π–π interactions with the ligand.

The GlideScore of the lead compounds were then compared with AR inhibitors epalrestat, sorbinil and ranirestat (S3 Fig). The results revealed that, in the three structures used in this study, the drugs epalrestat, sorbinil and ranirestat exhibited lower GlideScores than the natural compounds discussed above (Table 1). Remarkably, the phytochemicals discussed here showed a higher affinity for AR than drugs developed to target this protein. From Table 2 it is evident that in most cases, the identified natural compounds produced better MM-GBSA based binding energies due to more favorable coulombic interactions, lipophilic interations and van der Waals’ interactions when compared to current drugs. Other physicochemical parameters were also calculated to explore the potential of these molecules to be lead compounds.

### Pharmacokinetic properties of natural inhibitors and drugs

Since molecular docking analysis produced several promising leads, various physicochemical parameters of these compounds were calculated using Qikprop. This provides a computed value for various physical and chemical parameters and ensures that these are within the acceptable range for a drug molecule. Molecular weight, oral absorption, central nervous system activity, blood brain barrier penetrations, aqueous solubility, LogP, solvent accessible surface area (SASA) were calculated. Table 3 lists the values for these descriptors for the natural compounds and commercially developed drugs. Molecular weight and aqueous solubility values are in the recommended range. The values indicate that gingerenones A, B and C are much more soluble than sorbinil. The solvent accessible surface area (SASA) of the phytochemicals were typically higher than the drugs. Orally administered drugs must be properly absorbed in the intestine; here the analysis showed that gingerenones A, B and C have the highest oral absorption rate of 100% and quercetin showed the least absorption value of 52.68%. Due to safety concerns, blood brain barrier (BBB) penetration rate and central nervous system activity were also calculated. The brain is protected from systemic circulation by the BBB. All natural compounds discussed here were found to be highly CNS inactive whereas epalrestat and ranirestat showed minimal amount of CNS activity. Here all predicted compounds were BBB negative suggesting safe administrability. Thus the natural compounds showed better drug likeliness and pharmacokinetic properties than existing drugs and quercetin (natural AR inhibitor) indicating their potential as lead compounds to inhibit AR.

**Table 3:** Pharmacokinetic parameters of natural compounds and drugs.

Name	Molecular weight	Oral absorption	CNS activity	BBB partition coefficient	Aqueous solubility	LogP	SASA
Range	<500	>80%high <25%low	-2 inactive2 active	(-3.0 to 1.2)	(-6.5 to 0.5)	<5	300–1000
Gingerenone A	356.4	100	-2	-1.372	-5.117	3.757	659.968
Gingerenone B	386.4	100	-2	-1.437	-5.722	4.326	748.61
Quercetin	302.2	52.68	-2	-2.34	-4.043	0.383	514.629
Calebin A	360.4	75.156	-2	-2.494	-4.928	2.571	685.211
Lariciresinol	286.2	88.808	-2	-1.297	-4.655	2.545	621.894
Gingerenone C	326.3	100	-2	-1.482	-5.185	3.926	671.736
Epalrestat	319.3	84.701	-1	-0.945	-4.448	3.62	567.965
Sorbinil	236.2	83.38	0	-0.294	-2.331	1.132	390.557
Ranirestat	420.1	88.24	-1	-0.743	-4.301	2.945	555.515

### Molecular dynamics simulations of top scored docked complex

To study the dynamic interaction of AR with the docked phytochemicals, molecular dynamics (MD) simulations of the AR structure (PDB ID: 4GCA) complexed with gingerenones A and B were performed using Desmond. 100 ns simulations of these structures were performed to observe how the binding site adapts to the docked ligand. RMSD of the protein Cα atoms with respect to the initial structure in these simulations stabilized to under 2 Å indicating a stable conformation of the protein (S6 and S7 Figs). The simulations reach equilibrium in the first few nanoseconds. While the secondary structures were faithfully conserved during the entirety of the simulations, the loop region between residues 217–223 exhibited the most fluctuation in both simulations (S6 and S7 Figs).

**Fig 5:**
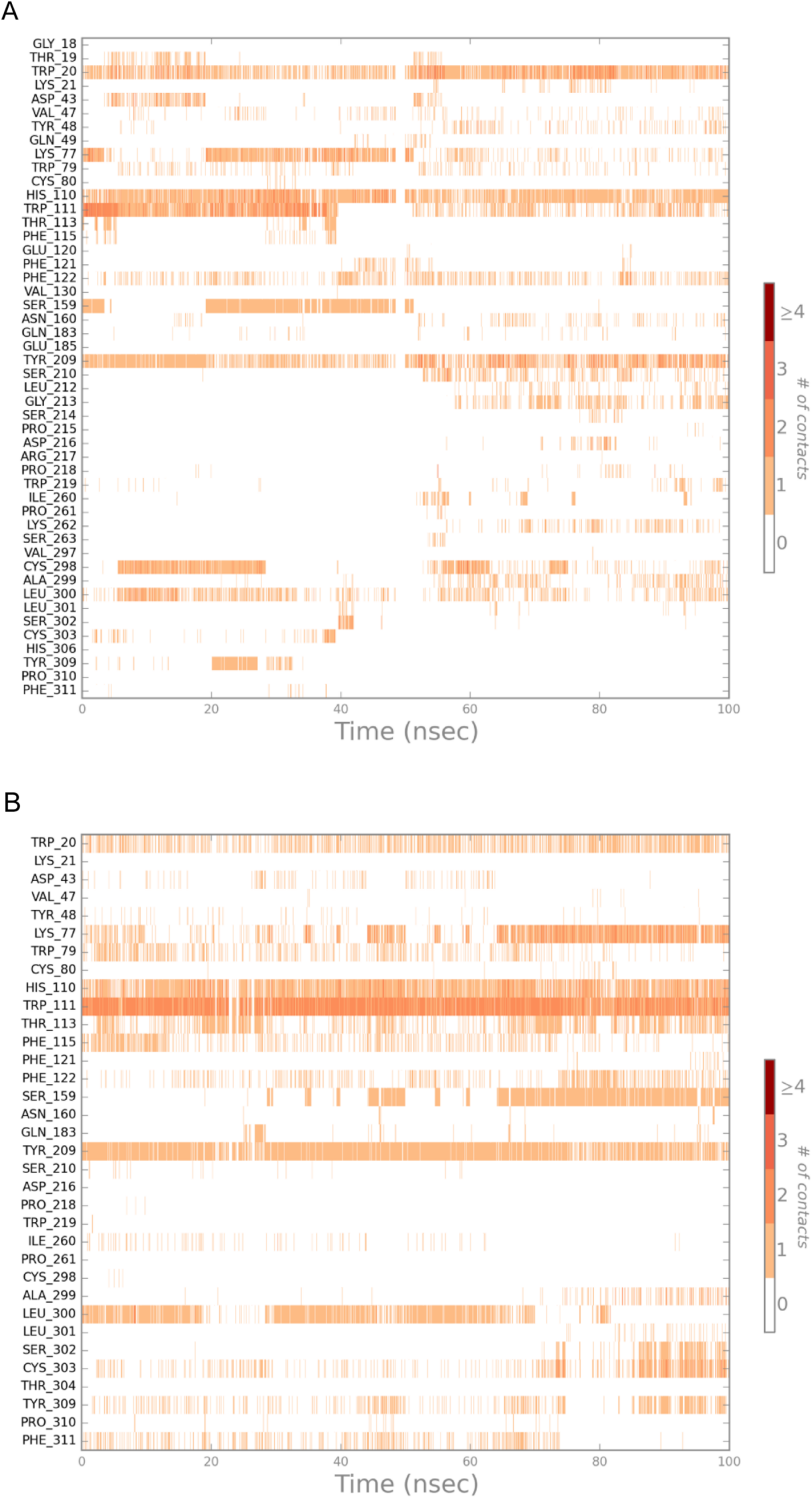
Residues of AR (PDB ID: 4GCA) that interact with the bound ligand and the number of interactions these residues make during the course of a 100 ns MD simulation. (A) AR-gingerenone A complex (B) AR-gingerenone B complex. The x-axis represents the MD simulation time at which the interaction was evaluated and the y-axis shows all the residues that interacted with ligand at some point during the simulation. The intensity of brown bar at any time point indicates how many contacts (hydrophobic, hydrogen bonds, etc.) a residue makes with the ligand at that time.

The binding site is a dynamic region and the simulations indicate that the protein structure is adapting around the docked ligand (Fig 5). Gingerenones A and B interacted with AR throughout the simulation by forming hydrogen bonds, hydrophobic interactions, π–π interactions and water bridges. In the AR-gingerenone A simulation, π–π interaction with the crucial His110 was maintained throughout, while the π–π interaction with Trp111 was intermittently broken. Interactions with the His110 and Trp111 in the anionic binding site of AR were maintained consistently in the AR-gingerenone B simulation. During the course of the simulation, new interactions were also developed between gingerenone A and Ser210, and gingerenone B and Lys77 and Ser159. These residues belong to the anionic binding and specificity region of the active site. Water molecules can also been observed to make transient contacts with the ligands and assists in the formation of bridged interactions with the protein.

Rescoring of the protein-ligand interactions was done using Glide after extracting frames every 5 ns from the equilibrium MD simulation. Ligand interaction diagrams depicting the protein-ligand interactions in the highest and the lowest scoring frames are shown in S8 and S9 Figs. In the case of gingerenone B, the average rescored GlideScore was -10.98 ± -1.92 kcal/mol along the equilibrium simlation with the final frame producing a GlideScore of -13.74 kcal/mol, which was a significant improvement on the initially docked GlideScore of -11.87 kcal/mol (Table 1). Gingerenone A simulation produced an average GlideScore of -12.41 ±1.43 kcal/mol and a slight improvement from -13.02 kcal/mol to -13.36 kcal/mol in the final frame of the simulation.

## Conclusion

Aldose reductase, the major rate limiting enzyme in the polyol pathway, plays a critical role in diabetic complications. Molecular docking revealed that natural compounds such as gingerenone A, gingerenone B, gingerenone C, quercetin, lariciresinol and calebin A from spices exhibited much better binding score and binding energy than commercially available drugs. MD simulations were performed to study how protein-ligand interactions evolved on a temporal scale. At the end of 100 ns simulations, rescoring of the protein-ligand interactions produced an improvement in the docking score due to novel interactions with proteins and water molecules. Thus, MD simulations could be an effective tool for refining protein-ligand interactions and obtaining a more accurate indication of the dynamic evolution of the binding site interactions. Molecular interactions and pharmacokinetic properties of these compounds are extremely favourable for developing new therapeutic strategies. Further *in vitro* and *in vivo* experimental studies are being considered for the validation of the results. Thus, this study sheds light on a mechanism through which these spices may have a positive effect for diabetics. The results further our understanding about how these natural molecules, present in our diet, may interact with proteins in our body.

## Supporting Information

S1 Table List of spices and phytochemicals used in this study. (DOCX)

S2 Table GlideScore after SP docking of phytocompounds and current drugs to AR. (DOCX)

S1 Fig Molecular interactions of lead molecules with AR (PDB ID: 4LAU). (A) AR-gingerenone A complex (B) AR-gingerenone B complex (C) AR-gingerenone C complex (D) AR-calebin A complex (E) AR-lariciresinol complex (F) AR-quercetin complex. Protein is shown in grey cartoon representation, amino acid side chains are shown in stick representation and the docked ligand is in orange. Hydrogen bonds are shown as black dotted lines and π–π interactions are shown as blue lines.
(TIF)

S2 Fig Molecular interactions of lead molecules with AR (PDB ID: 1US0). (A) AR-gingerenone A complex (B) AR-gingerenone B complex (C) AR-gingerenone C complex (D) AR-calebin A complex (E) AR-lariciresinol complex (F) AR-quercetin complex. Protein is shown in grey cartoon representation, amino acid side chains are shown in stick representation and the docked ligand is in orange. Hydrogen bonds are shown as black dotted lines and π–π interactions are shown as blue lines.
(TIF)

S3 Fig Molecular interactions of drugs with AR (PDB ID: 4GCA). (A) AR-epalrestat complex (B) AR-ranirestat complex (C) AR-sorbinil complex. Protein is shown in grey cartoon representation, amino acid side chains are shown in stick representation and the docked ligand is in orange. Hydrogen bonds are shown as black dotted lines and π–π interactions are shown as blue lines.
(TIF)

S4 Fig Ligand interaction diagrams of lead compounds with AR (PDB ID: 4LAU). (A) AR-gingerenone A complex (B) AR-gingerenone B complex (C) AR-gingerenone C complex (D) AR-calebin A complex (E) AR-lariciresinol complex (F) AR-quercetin complex. Colored circles indicate amino acids that interact with the bound ligand. Negatively charged amino acids are represented with red circles, positively charged amino acids are represented with dark blue circles, polar amino acids are represented with light blue circles and hydrophobic amino acids are represented with green circles. Hydrogen bonds are represented with purple arrows–dashed arrows for hydrogen bonds involving amino acid side chain and regular arrows for hydrogen bonds involving amino acid backbone. π–π interactions are shown with green lines.
(TIF)

S5 Fig Ligand interaction diagrams of lead compounds with AR (PDB ID: 1US0). (A) AR-gingerenone A complex (B) AR-gingerenone B complex (C) AR-gingerenone C complex (D) AR-calebin A complex (E) AR-lariciresinol complex (F) AR-quercetin complex. Colored circles indicate amino acids that interact with the bound ligand. Negatively charged amino acids are represented with red circles, positively charged amino acids are represented with dark blue circles, polar amino acids are represented with light blue circles and hydrophobic amino acids are represented with green circles. Hydrogen bonds are represented with purple arrows–dashed arrows for hydrogen bonds involving amino acid side chain and regular arrows for hydrogen bonds involving amino acid backbone. π–π interactions are shown with green lines.
(TIF)

S6 Fig RMSD and RMSF from MD simulation of AR (PDB ID: 4GCA) with gingerenone A. (A) RMSD of Cα atoms of AR with respect to the initial structure during the course of the simulation. Simulation reaches equilibrium in the first few nanoseconds as indicated by the plateauing of the RMSD plot. (B) RMSF of Cα atoms of AR indicating backbone regions with major motions. Significant movement is observed in the loop region between residues 217–223.
(TIF)

S7 Fig RMSD and RMSF from MD simulation of AR (PDB ID: 4GCA) with gingerenone B. (A) RMSD of Cα atoms of AR with respect to the initial structure during the course of the simulation. Simulation reaches equilibrium in the first few nanoseconds as indicated by the plateauing of the RMSD plot. (B) RMSF of Cα atoms of AR indicating backbone regions with major motions. Significant movement is observed in the loop region between residues 217–223.
(TIF)

S8 Fig Ligand interaction diagrams from frames of the 4GCA-gingerenone A MD simulation. The top 3 highest and lowest scoring frames are shown along with the corresponding rescored GlideScore. Colored circles indicate amino acids that interact with the bound ligand. Negatively charged amino acids are represented with red circles, positively charged amino acids are represented with dark blue circles, polar amino acids are represented with light blue circles and hydrophobic amino acids are represented with green circles. Water molecules are represented with gray circles. Hydrogen bonds are represented with purple arrows–dashed arrows for hydrogen bonds involving amino acid side chain and regular arrows for hydrogen bonds involving amino acid backbone. π–π interactions are shown with green lines.
(TIF)

S9 Fig Ligand interaction diagrams from frames of the 4GCA-gingerenone B MD simulation. The top 3 highest and lowest scoring frames are shown along with the corresponding rescored GlideScore. Colored circles indicate amino acids that interact with the bound ligand. Negatively charged amino acids are represented with red circles, positively charged amino acids are represented with dark blue circles, polar amino acids are represented with light blue circles and hydrophobic amino acids are represented with green circles. Water molecules are represented with gray circles. Hydrogen bonds are represented with purple arrows–dashed arrows for hydrogen bonds involving amino acid side chain and regular arrows for hydrogen bonds involving amino acid backbone. π–π interactions are shown with green lines.
(TIF)
