# Supplementary material for: Identification of Novel Aldose Reductase Inhibitors from Spices: A Molecular Docking and Simulation Study
Source: PLoS One. 2015 Sep 18;10(9):e0138186. doi: 10.1371/journal.pone.0138186 (PMC4575143; doi:10.1371/journal.pone.0138186)
Supplement: S1 Table — (DOCX) [file pone.0138186.s001.docx]

**S1 Table. List of spices and phytochemicals used in this study**

| **No:** | **PHYTOCHEMICAL** | **PUBCHEM ID** |
| --- | --- | --- |
| ***Allium sativum* (garlic)** | | |
|  | 2,3,4-trithiapentane | 19310 |
|  | Allixin | 86374 |
|  | Kaempferol | 5280863 |
|  | Allyl methyl trisulphide | 61926 |
|  | 2-methylbenzaldehyde | 10722 |
|  | Allyl methyl disulfide | 62434 |
|  | Alliin | 9576089 |
|  | Beta carotene | 5280489 |
|  | Allyl alcohol | 7858 |
|  | Diallyl disulfide | 16590 |
|  | Propylene sulfide | 14072 |
|  | Allyl sulfide | 11617 |
|  | 2-vinyl-4h-1,3-dithiin | 133337 |
|  | Allyl propyl disulfide | 16591 |
|  | 1,3-dithiane | 10451 |
|  | Diallyl trisulfide | 16315 |
|  | Ajoene | 5386591 |
|  | Alpha phellandrene | 7460 |
|  | 1-hexanol | 8103 |
|  | Quercetin | 5280343 |
|  | Allicin | 65036 |
|  | Brovanexine | 193294 |
|  | 3,5-diethyl-1,2,4-trithiolane | 520895 |
|  | S-allyl cysteine | 57461954 |
|  | Alliin | 87310 |
|  | Diallyl tetrasulfide | 75552 |
|  | 4-methyl-5-vinylthiazole | 15654 |
| ***Curcuma longa* (turmeric)** | | |
|  | (1,2,3-trimethyl-cyclopent-2-enyl)-methanol | 572946 |
|  | Alpha-atlantone | 558173 |
|  | Beta-bisabolene | 10104370 |
|  | Caryophyllene oxide | 1742210 |
|  | 2,2,4-trimethyl-3-(3,8,12,16-tetramethyl-heptadeca-3,7,11,15-tetraenyl)-cyclohexanol | 5366008 |
|  | Alpha-bergamotene | 6428986 |
|  | Beta-curcumene | 14014430 |
|  | Caryophyllene | 5281515 |
|  | 2,3,5-trimethylfuran | 517859 |
|  | Bisabolol | 1549992 |
|  | Beta-elemene | 6918391 |
|  | 2,4-dimethyl-8-oxabicyclo[3.2.1]oct-6-en-3-one | 557211 |
|  | Beta-myrcene | 31253 |
|  | Farnesol | 445070 |
|  | Alpha santalene | 94164 |
|  | Beta-phellandrene | 11142 |
|  | 2-carene | 79044 |
|  | Alpha-santalol | 6857559 |
|  | Beta-pinene | 14896 |
|  | 3-carene | 26049 |
|  | Alpha selinene | 10123 |
|  | Alpha-santalene | 10534 |
|  | 4,5-dimethyl-2,6-octadiene | 519579 |
|  | gamma-terpinene | 7462 |
|  | Beta-sesquiphellandrene | 12315492 |
|  | 4,8-dimethyl-3,7-nonadien-2-ol | 5365818 |
|  | Alpha-terpineol | 17100 |
|  | Beta-turmerone | 196216 |
|  | Dehydrozingerone | 5354238 |
|  | Alpha-thujene | 12444322 |
|  | 2,4-dimethyl-3-nitrobicyclo[3.3.1]nonan-9-one | 557173 |
|  | (4s,5s)-(+)-germacrone 4,5-epoxide | 13922653 |
|  | Zingiberene | 92776 |
|  | Bisabolone | 6429340 |
|  | Nerylacetone | 1713001 |
|  | Aristolene | 530421 |
|  | Isoborneol | 64685 |
|  | 7-epi-sesquithujene | 56927990 |
|  | Ar-turmerone | 160512 |
|  | Bornyl acetate | 6448 |
|  | Acoradiene | 90351 |
|  | Ascaridole | 10545 |
|  | Calebin-A | 637429 |
|  | Adoxal | 98403 |
|  | Benzene, 1-methyl-4-(1-methylpropyl) | 519195 |
|  | Camphene | 6616 |
|  | Humulene | 5281520 |
|  | Gamma-sitosterol | 222284 |
|  | Camphor | 2537 |
|  | Alpha-pinene | 6654 |
|  | (2-methylpropenyl)benzene | 13030 |
|  | D-carvone | 16724 |
|  | Alpha-curcumene | 92139 |
|  | Carvacrol | 10364 |
|  | (e,e,e)-3,7,11,15-tetramethylhexadeca-1,3,6,10,14-pentaene | 5365883 |
|  | Menthofuran | 329983 |
|  | Stigmasterol | 5280794 |
|  | Chrysanthenyl acetate | 162747 |
|  | Procurcumenol | 189061 |
|  | Menthol | 16666 |
|  | Cyclohexene | 12304570 |
|  | Eucalyptol | 2758 |
|  | Ferulic acid | 445858 |
|  | Methyleugenol | 7127 |
|  | Teresantalol | 578221 |
|  | (z)-cinerone | 5373127 |
|  | (e)-gamma-bisabolene | 5352437 |
|  | Citral | 643779 |
|  | Terpinenol-4 | 11230 |
|  | Citronellal | 7794 |
|  | Gamma-curcumene | 12304273 |
|  | Geraniol | 643820 |
|  | Terpinolene | 11463 |
|  | Citronellyl valerate | 61416 |
|  | Gamma elemene | 6432312 |
|  | Nerolidyl propionate | 5365021 |
|  | Thymol | 6989 |
|  | Corymbolone | 535226 |
|  | Gamma-terpinene | 7461 |
|  | Geraniol acetate | 7780 |
|  | Vanillic acid | 8468 |
|  | 6-cubebene | 93081 |
|  | Gamma-terpineol | 11467 |
|  | 1-methyl-2-isopropylbenzene | 10703 |
|  | Vanillin | 1183 |
|  | Curcumin | 969516 |
|  | Oleic acid | 445639 |
|  | Xanthorrhizol | 93135 |
|  | Curcumenol | 12304275 |
|  | Decaprenoic acid | 5275520 |
|  | Palmitic acid | 985 |
|  | Ferulic acid | 1548883 |
|  | Curcumenone | 42608155 |
|  | Geraniol | 637566 |
|  | P-cymen-8-ol | 14529 |
|  | Zingerone | 31211 |
|  | Geranyl acetate | 1549026 |
|  | 4-cymene | 7463 |
|  | Diferuloylmethane | 969516 |
|  | Germacrene d | 5317570 |
|  | Phytol | 5280435 |
|  | Demethoxycurcumin | 5469424 |
|  | Germacrone | 6436348 |
|  | D-piperitone | 61362 |
|  | Bisdemethoxy curcumin | 5315472 |
|  | Gitoxigenin | 348482 |
|  | Dihydrocarvone | 24473 |
|  | Curcuphenol | 360253 |
|  | Himachalene | 15095 |
|  | Terpinolene | 11463 |
|  | Cyclohexyl formate | 20358 |
|  | Lupeol | 259846 |
|  | P-methylacetophenone | 8500 |
|  | Dehydrocurdione | 10421549 |
|  | Limonene | 440917 |
|  | Procurcumadiol | 14633012 |
|  | Dehydrozingerone | 5354238 |
|  | Linalool | 6549 |
|  | Procurcumenol | 189061 |
|  | Dicumyl peroxide | 6641 |
|  | Linoleic acid | 5280450 |
|  | Pyrazolo[1,5-a]pyridine, 3,3a,4,7-tetrahydro-3,3-dimethyl-, (3as)-, 3,3-dimethyl-4,7-dihydro-3ah-pyrazolo[1,5-a]pyridine | 591596 |
|  | Alpha-farnesene | 5281516 |
|  | 1-methyl-3-isopropylbenzene | 10812 |
|  | Stearic acid | 5281 |
| ***Zingiber officinale* (ginger)** | | |
|  | Cyclosativene | 16212927 |
|  | 8-paradol | 213821 |
|  | Zingiberene | 92776 |
|  | Isoborneol | 64685 |
|  | Norcamphor | 10345 |
|  | 8-gingerol | 168114 |
|  | Beta-bisabolene | 10104370 |
|  | Gingerdiol | 11369949 |
|  | (8)-shogaol | 6442560 |
|  | Beta bisabolol | 27208 |
|  | Gingerdione | 162952 |
|  | Gingerol | 168115 |
|  | Beta-farnesene | 5281517 |
|  | Gingerol | 442793 |
|  | (10)-shogaol | 6442612 |
|  | Beta-phellandrene | 11142 |
|  | 6-paradol | 94378 |
|  | Alpha-curcumene | 92139 |
|  | Beta pinene, 3-(acetylmethyl) | 539536 |
|  | Shogaol | 5281794 |
|  | Alpha-farnesene | 5281516 |
|  | Sesquiphellandrene | 519764 |
|  | Camphene | 6616 |
|  | Gamma-tocopherol | 92729 |
|  | Limonene | 22311 |
|  | 1,8-dimethyl-4-(1-methylethenyl)spiro(4.5)dec-7-ene | 90351 |
|  | Capsaicin | 1548943 |
|  | Geraniol | 637566 |
|  | Linalool | 6549 |
|  | Widdrol | 94334 |
|  | Carveol | 7438 |
|  | Geranyl acetate | 1549026 |
|  | Matairesinol | 119205 |
|  | Eucalyptol | 2758 |
|  | Germacrone | 6436348 |
|  | Methyl linolenate | 5319706 |
|  | Gingerenone A | 5281775 |
|  | Gingerenone B | 5317592 |
|  | Gingerenone C | 5317593 |
|  | Naphthalene | 931 |
|  | Farnesene epoxide, e- | 5362910 |
|  | Guaiol | 227829 |
|  | Nerolidyl acetate | 5363426 |
|  | Gamma-cadinene | 92313 |
|  | Lariciresinol | 332427 |
|  | Nerolidol | 5284507 |
| ***Trigonella foenum graecum* (fenugreek)** | | |
|  | Pyrrole-3-carboxylic acid | 101030 |
|  | Diosgenin | 99474 |
|  | 3,3-dimethylindolin-2-one | 313100 |
|  | Gitogenin | 441887 |
|  | Allylamine | 7853 |
|  | Harmaline | 5280951 |
|  | 3-O-methylglucose | 8973 |
|  | Glycolic acid | 757 |
|  | Quararibea lactone | 354197 |
|  | Methyl nicotinate | 7151 |
|  | Hexopyranose | 79064 |
|  | Sarsasapogenin | 219836 |
|  | Aziridine | 9033 |
|  | Trigonelline hydrochloride | 134606 |
