# Supplementary material for: Identification of Novel Aldose Reductase Inhibitors from Spices: A Molecular Docking and Simulation Study
Source: PLoS One. 2015 Sep 18;10(9):e0138186. doi: 10.1371/journal.pone.0138186 (PMC4575143; doi:10.1371/journal.pone.0138186)
Supplement: S2 Table — (DOCX) [file pone.0138186.s002.docx]

**S2 Table. GlideScore after SP docking of phytocompounds and current drugs to AR**

| **Target** | **Ligand** | **SP GlideScore (kcal/mol)** |
| --- | --- | --- |
| 4GCA | Gingerenone A | -9.02 |
| 4GCA | Gingerenone B | -6.12 |
| 4GCA | Quercetin | -7.85 |
| 4GCA | Lariciresinol | -9.47 |
| 4GCA | Calebin A | -9.2 |
| 4GCA | Gingerenone C | -9.21 |
| 4GCA | Ranirestat | -7.89 |
| 4GCA | Epalrestat | -9.76 |
| 4GCA | Sorbinil | -7.4 |
| 4LAU | Gingerenone B | -7.87 |
| 4LAU | Gingerenone A | -8.8 |
| 4LAU | Quercetin | -8.6 |
| 4LAU | Calebin A | -7.27 |
| 4LAU | Lariciresinol | -9.09 |
| 4LAU | Gingerenone C | -9.14 |
| 4LAU | Epalrestat | -9.6 |
| 4LAU | Ranirestat | -9.5 |
| 4LAU | Sorbinil | -7.12 |
| 1US0 | Gingerenone B | -7.84 |
| 1US0 | Gingerenone A | -8.9 |
| 1US0 | Quercetin | -8.92 |
| 1US0 | Gingerenone C | -9.24 |
| 1US0 | Lariciresinol | -8.91 |
| 1US0 | Calebin A | -6.49 |
| 1US0 | Ranirestat | -9.35 |
| 1US0 | Epalrestat | -9.39 |
| 1US0 | Sorbinil | -8.35 |
