# Supplementary figures and images for: Identification of Novel Aldose Reductase Inhibitors from Spices: A Molecular Docking and Simulation Study
Source: PLoS One. 2015 Sep 18;10(9):e0138186. doi: 10.1371/journal.pone.0138186 (PMC4575143; doi:10.1371/journal.pone.0138186)

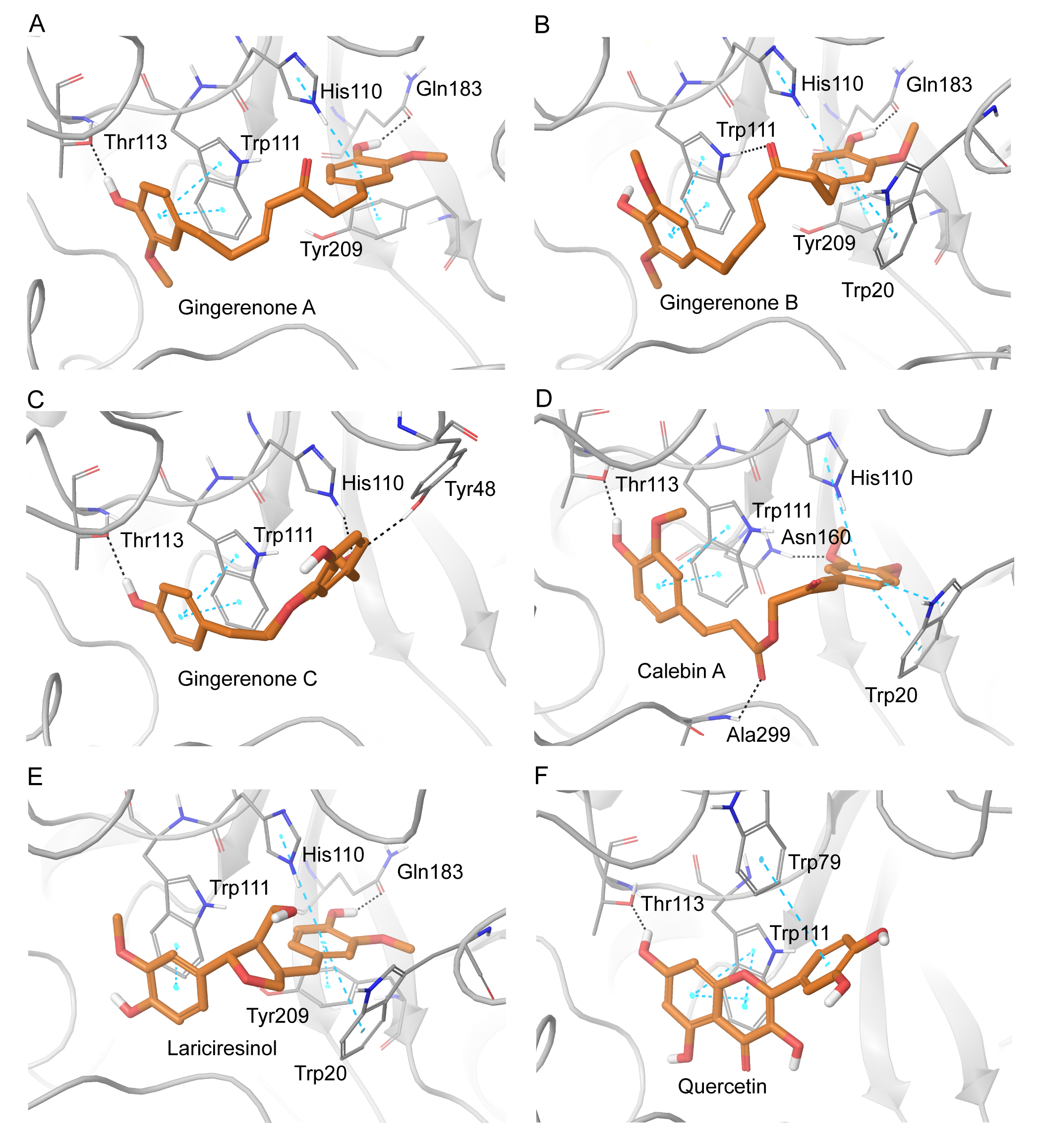

Supplement: S1 Fig — (A) AR-gingerenone A complex (B) AR-gingerenone B complex (C) AR-gingerenone C complex (D) AR-calebin A complex (E) AR-lariciresinol complex (F) AR-quercetin complex. Protein is shown in grey cartoon representation, amino acid side chains are shown in stick representation and the docked ligand is in orange. Hydrogen bonds are shown as black dotted lines and π–π interactions are shown as blue lines. (TIF) [file pone.0138186.s003.tif]

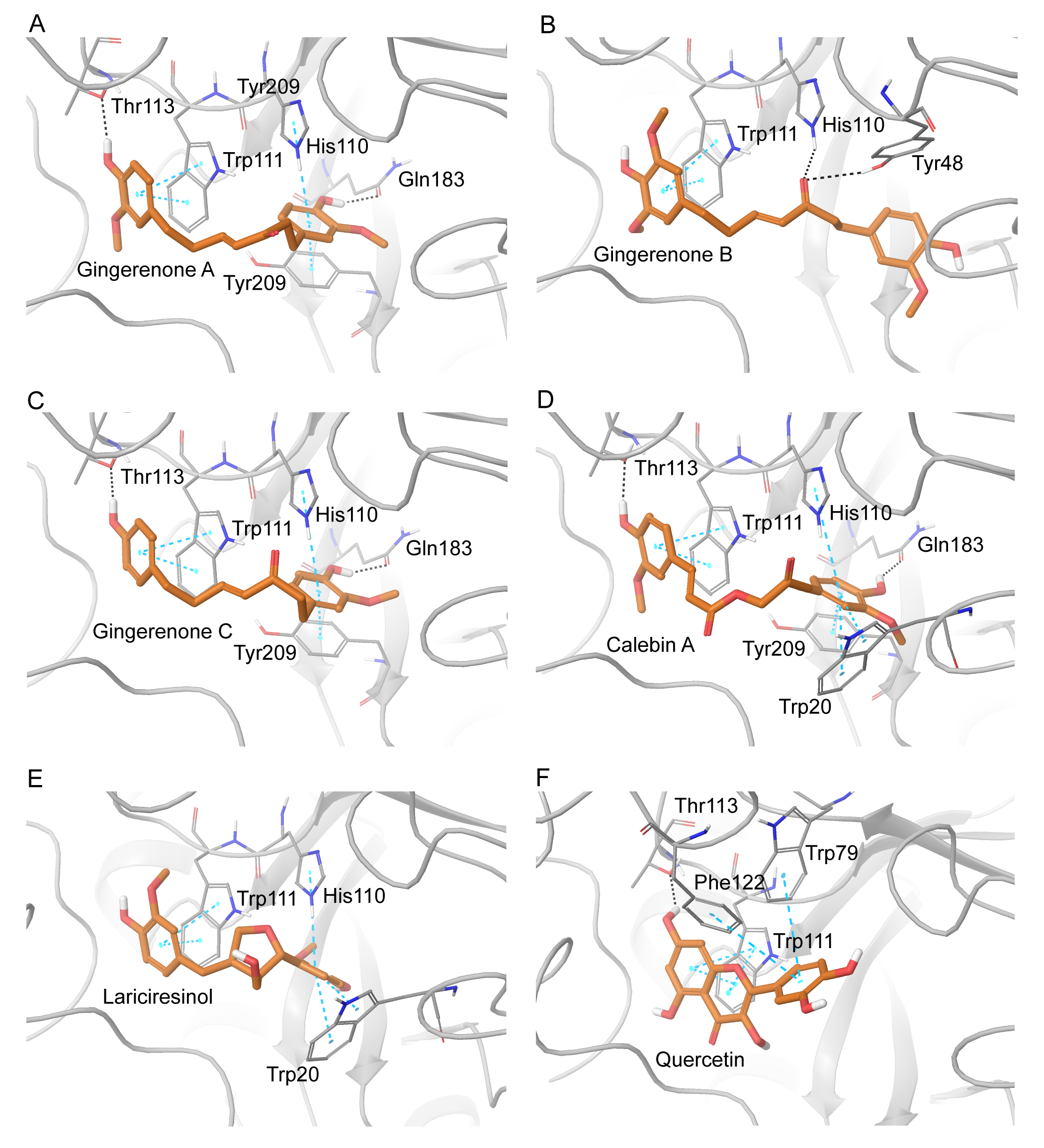

Supplement: S2 Fig — (A) AR-gingerenone A complex (B) AR-gingerenone B complex (C) AR-gingerenone C complex (D) AR-calebin A complex (E) AR-lariciresinol complex (F) AR-quercetin complex. Protein is shown in grey cartoon representation, amino acid side chains are shown in stick representation and the docked ligand is in orange. Hydrogen bonds are shown as black dotted lines and π–π interactions are shown as blue lines. (TIF) [file pone.0138186.s004.tif]

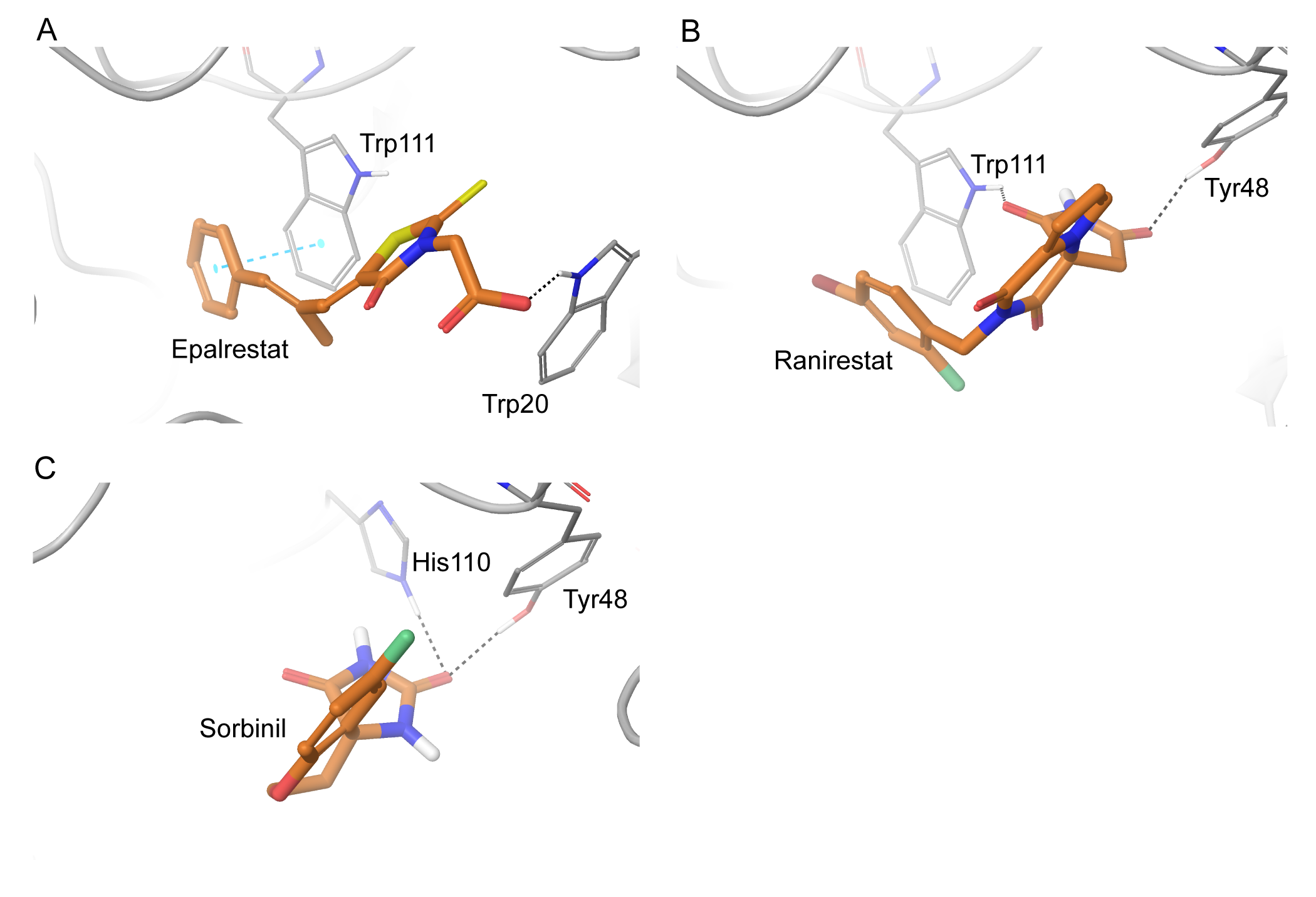

Supplement: S3 Fig — (A) AR-epalrestat complex (B) AR-ranirestat complex (C) AR-sorbinil complex. Protein is shown in grey cartoon representation, amino acid side chains are shown in stick representation and the docked ligand is in orange. Hydrogen bonds are shown as black dotted lines and π–π interactions are shown as blue lines. (TIF) [file pone.0138186.s005.tif]

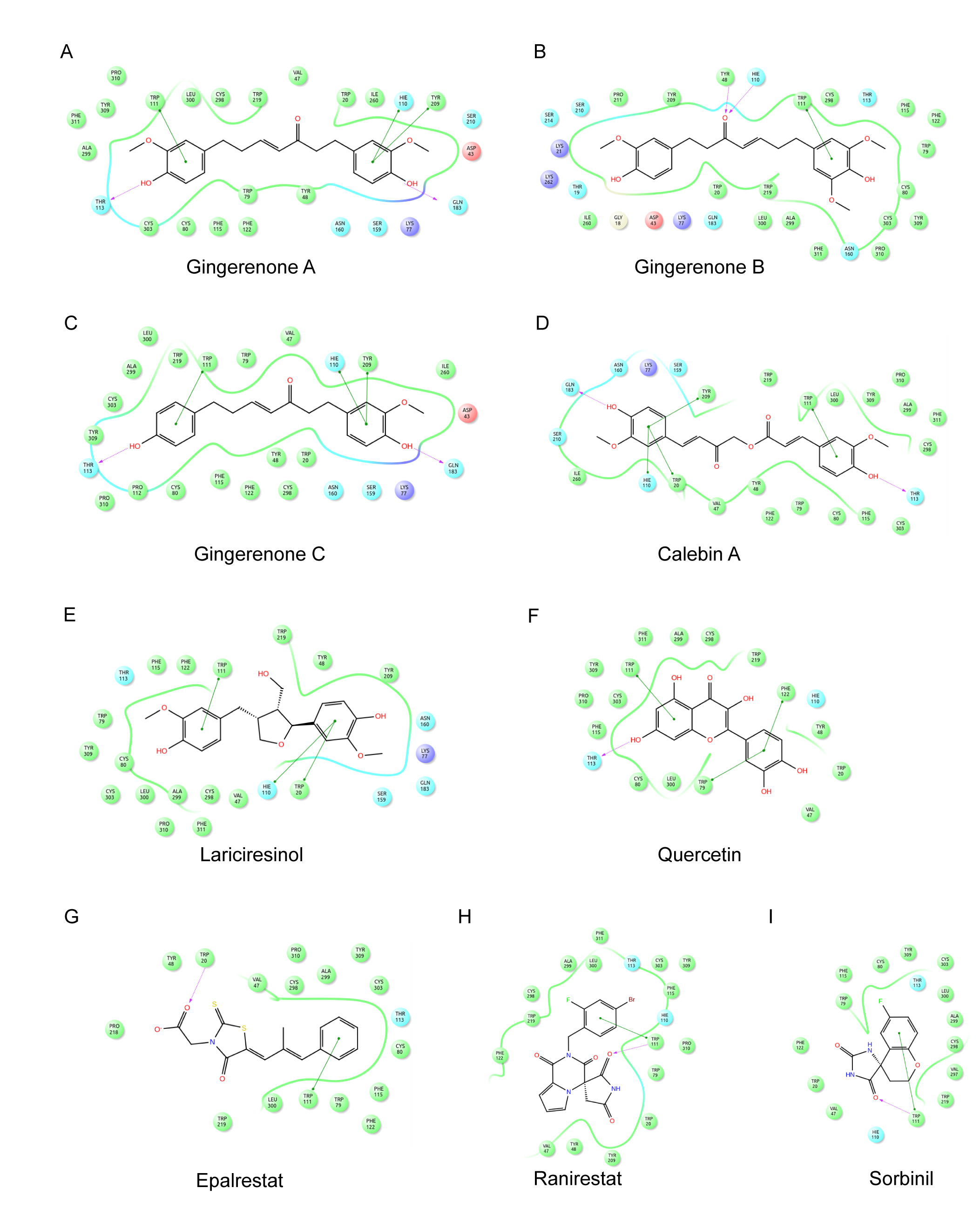

Supplement: S4 Fig — (A) AR-gingerenone A complex (B) AR-gingerenone B complex (C) AR-gingerenone C complex (D) AR-calebin A complex (E) AR-lariciresinol complex (F) AR-quercetin complex. Colored circles indicate amino acids that interact with the bound ligand. Negatively charged amino acids are represented with red circles, positively charged amino acids are represented with dark blue circles, polar amino acids are represented with light blue circles and hydrophobic amino acids are represented with green circles. Hydrogen bonds are represented with purple arrows–dashed arrows for hydrogen bonds involving amino acid side chain and regular arrows for hydrogen bonds involving amino acid backbone. π–π interactions are shown with green lines. (TIF) [file pone.0138186.s006.tif]

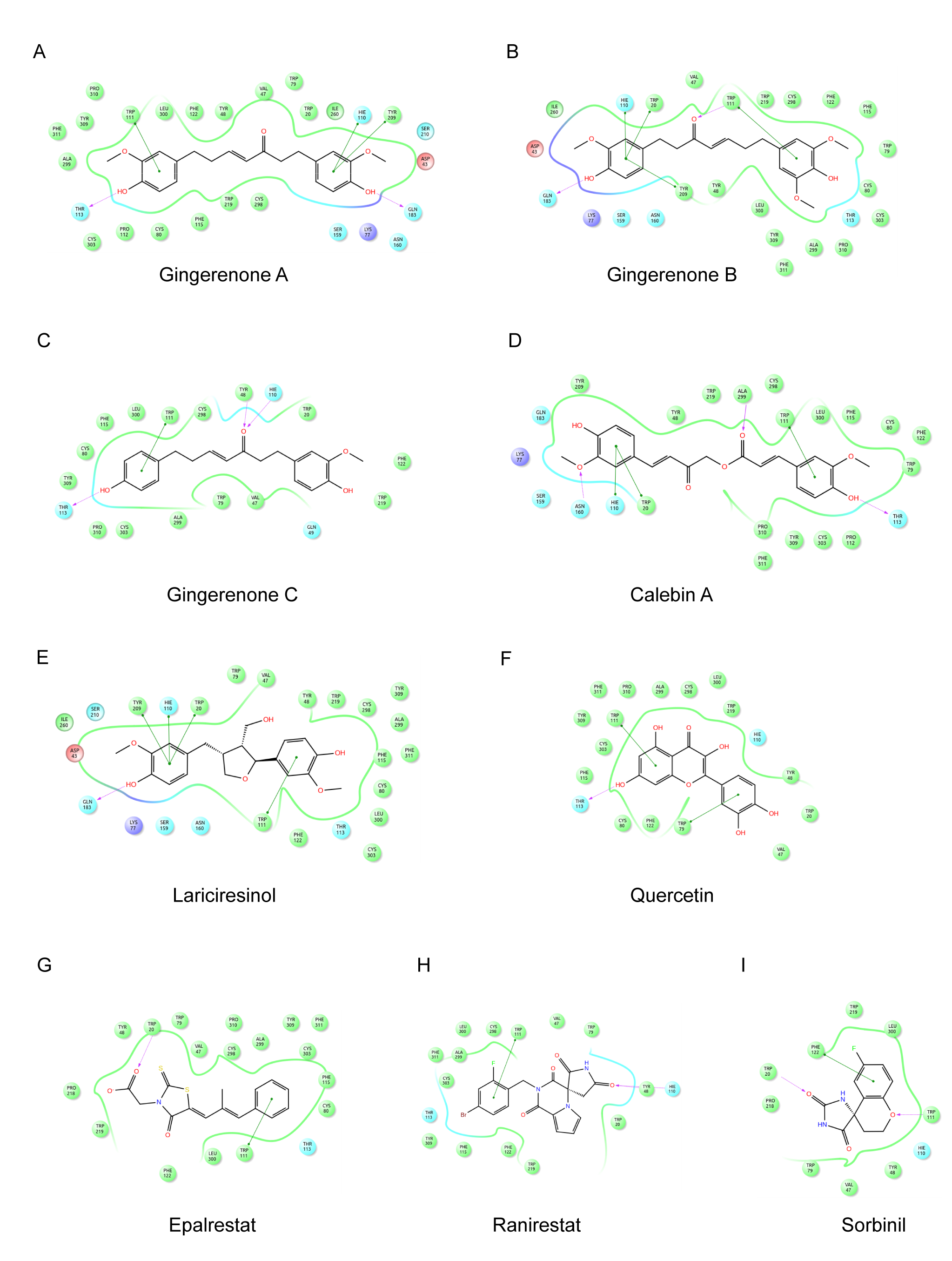

Supplement: S5 Fig — (A) AR-gingerenone A complex (B) AR-gingerenone B complex (C) AR-gingerenone C complex (D) AR-calebin A complex (E) AR-lariciresinol complex (F) AR-quercetin complex. Colored circles indicate amino acids that interact with the bound ligand. Negatively charged amino acids are represented with red circles, positively charged amino acids are represented with dark blue circles, polar amino acids are represented with light blue circles and hydrophobic amino acids are represented with green circles. Hydrogen bonds are represented with purple arrows–dashed arrows for hydrogen bonds involving amino acid side chain and regular arrows for hydrogen bonds involving amino acid backbone. π–π interactions are shown with green lines. (TIF) [file pone.0138186.s007.tif]

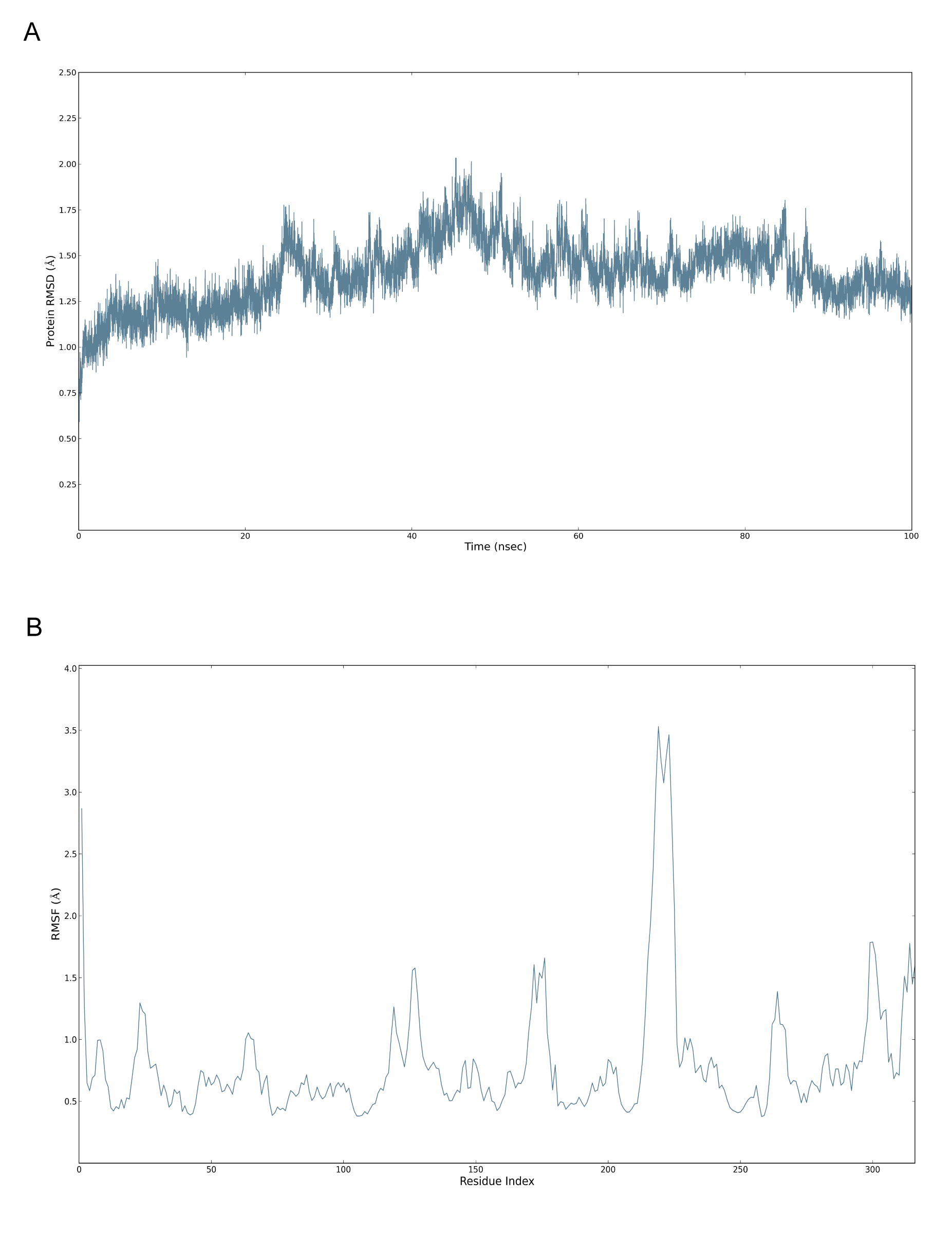

Supplement: S6 Fig — (A) RMSD of Cα atoms of AR with respect to the initial structure during the course of the simulation. Simulation reaches equilibrium in the first few nanoseconds as indicated by the plateauing of the RMSD plot. (B) RMSF of Cα atoms of AR indicating backbone regions with major motions. Significant movement is observed in the loop region between residues 217–223. (TIF) [file pone.0138186.s008.tif]

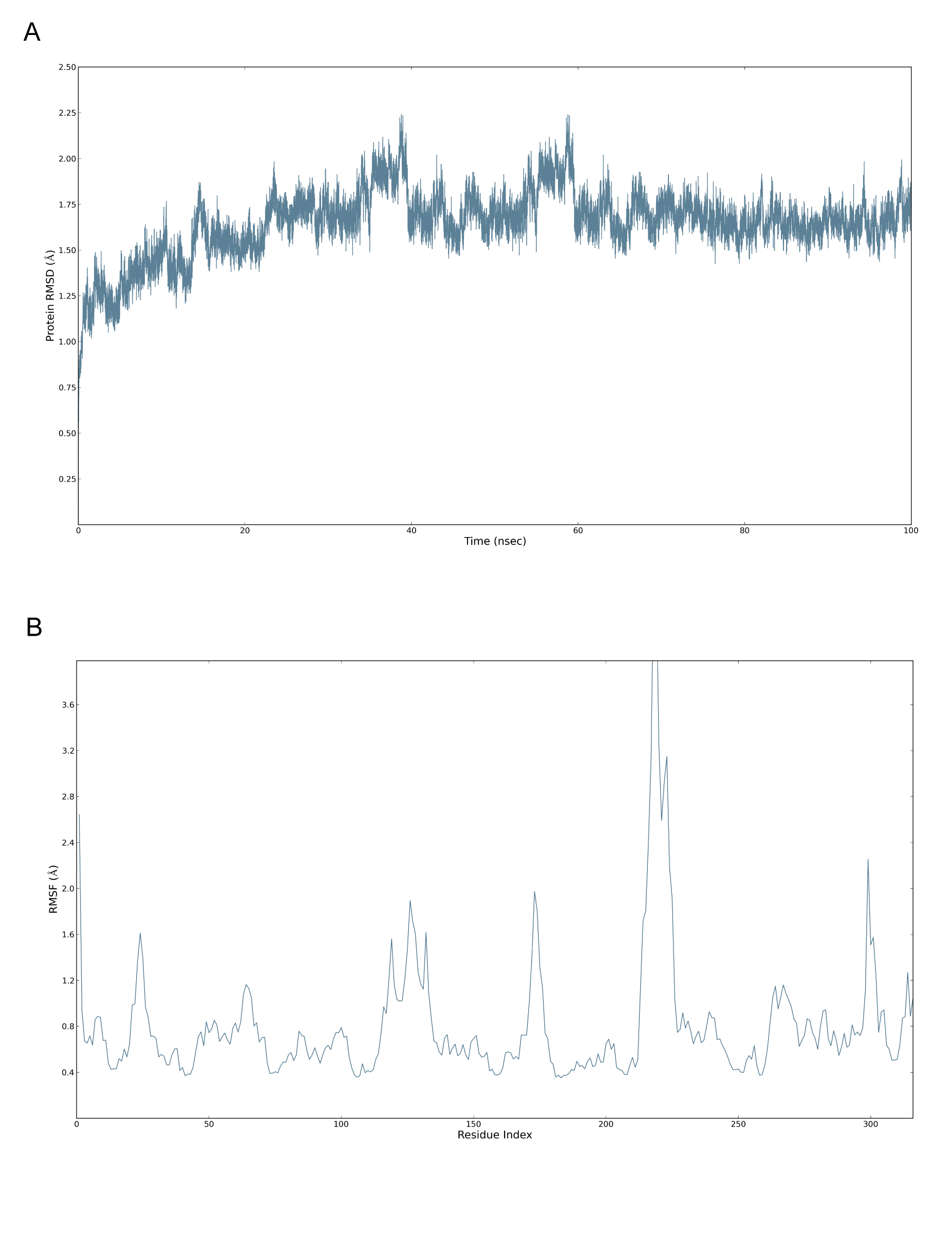

Supplement: S7 Fig — (A) RMSD of Cα atoms of AR with respect to the initial structure during the course of the simulation. Simulation reaches equilibrium in the first few nanoseconds as indicated by the plateauing of the RMSD plot. (B) RMSF of Cα atoms of AR indicating backbone regions with major motions. Significant movement is observed in the loop region between residues 217–223. (TIF) [file pone.0138186.s009.tif]

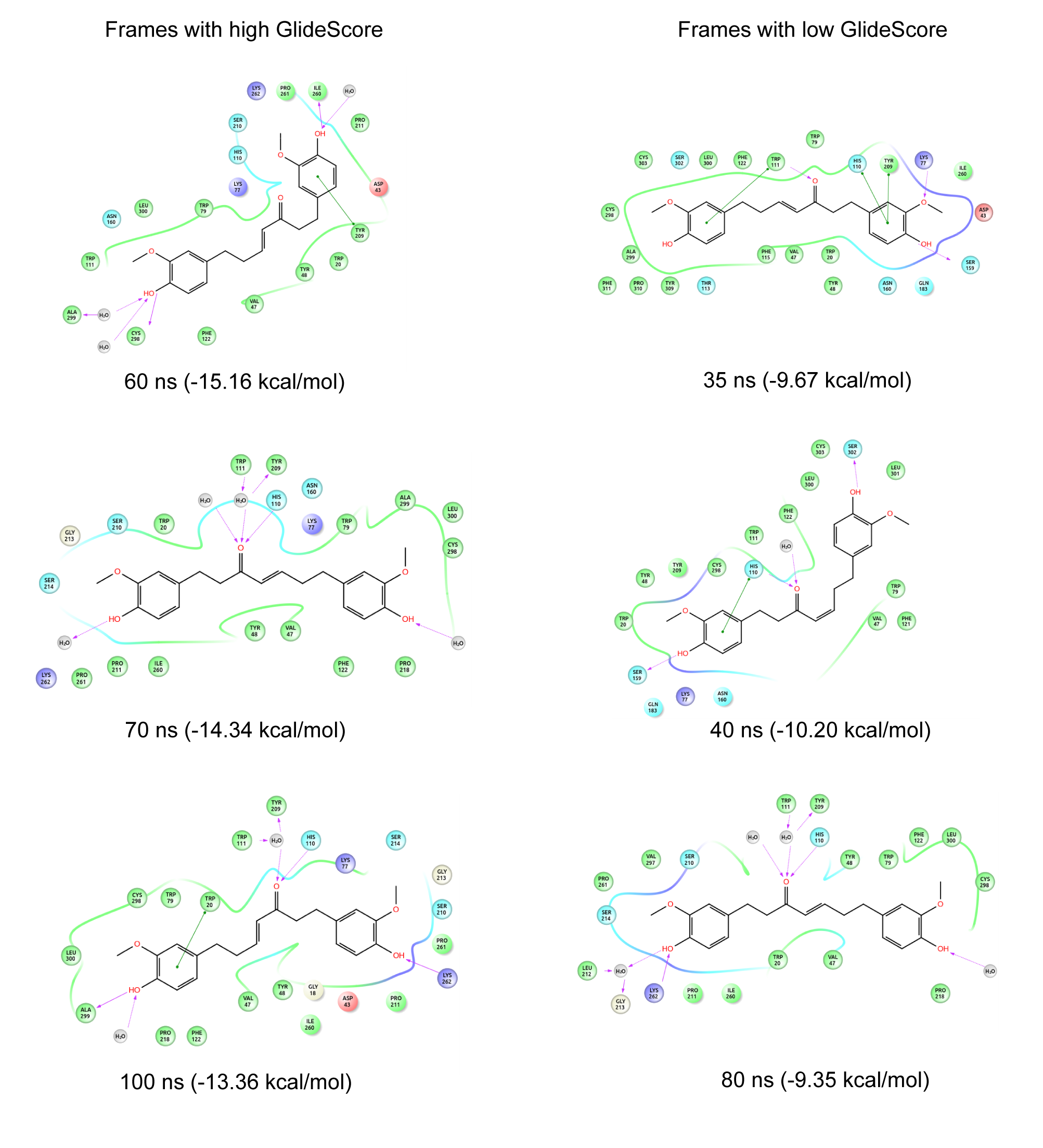

Supplement: S8 Fig — The top 3 highest and lowest scoring frames are shown along with the corresponding rescored GlideScore. Colored circles indicate amino acids that interact with the bound ligand. Negatively charged amino acids are represented with red circles, positively charged amino acids are represented with dark blue circles, polar amino acids are represented with light blue circles and hydrophobic amino acids are represented with green circles. Water molecules are represented with gray circles. Hydrogen bonds are represented with purple arrows–dashed arrows for hydrogen bonds involving amino acid side chain and regular arrows for hydrogen bonds involving amino acid backbone. π–π interactions are shown with green lines. (TIF) [file pone.0138186.s010.tif]

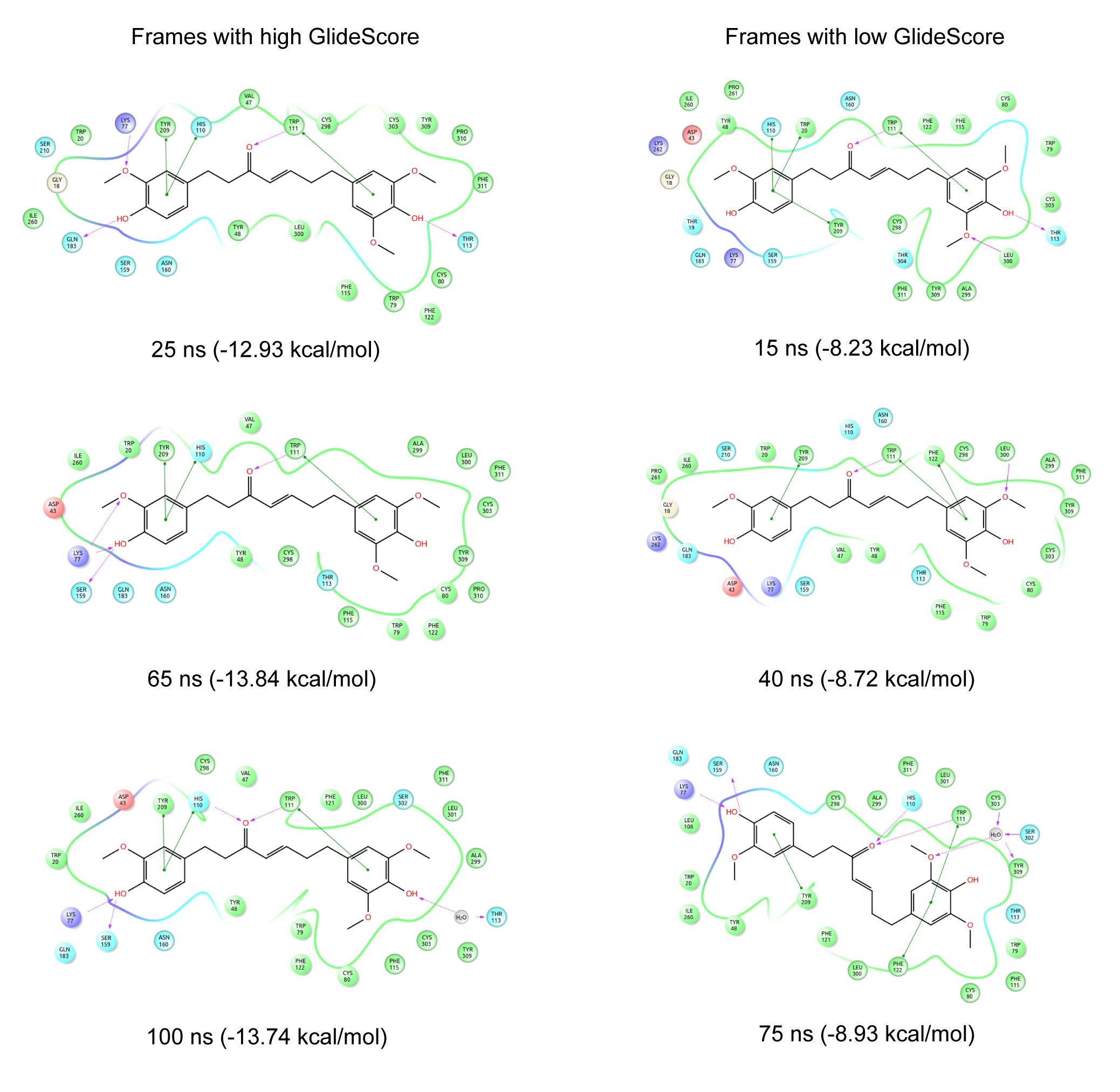

Supplement: S9 Fig — The top 3 highest and lowest scoring frames are shown along with the corresponding rescored GlideScore. Colored circles indicate amino acids that interact with the bound ligand. Negatively charged amino acids are represented with red circles, positively charged amino acids are represented with dark blue circles, polar amino acids are represented with light blue circles and hydrophobic amino acids are represented with green circles. Water molecules are represented with gray circles. Hydrogen bonds are represented with purple arrows–dashed arrows for hydrogen bonds involving amino acid side chain and regular arrows for hydrogen bonds involving amino acid backbone. π–π interactions are shown with green lines. (TIF) [file pone.0138186.s011.tif]
